# Halloysite/Keratin Nanocomposite for Human Hair Photoprotection Coating

**DOI:** 10.1021/acsami.0c05252

**Published:** 2020-05-06

**Authors:** Giuseppe Cavallaro, Stefana Milioto, Svetlana Konnova, Gölnur Fakhrullina, Farida Akhatova, Giuseppe Lazzara, Rawil Fakhrullin, Yuri Lvov

**Affiliations:** † Dipartimento di Fisica e Chimica, Università degli Studi di Palermo, Viale delle Scienze, pad. 17, Palermo 90128, Italy; ‡ Consorzio Interuniversitario Nazionale per la Scienza e Tecnologia dei Materiali, INSTM, Via G. Giusti, 9, Firenze I-50121, Italy; § Institute of Fundamental Medicine and Biology, Kazan Federal University, Kreml uramı 18, Kazan, Republic of Tatarstan 420008, Russian Federation; ∥ Institute for Micromanufacturing, Louisiana Tech University, 505 Tech Drive, Ruston, Louisiana 71272, United States

**Keywords:** halloysite nanotubes, keratin, composite, hair treatment, UV-protective coating

## Abstract

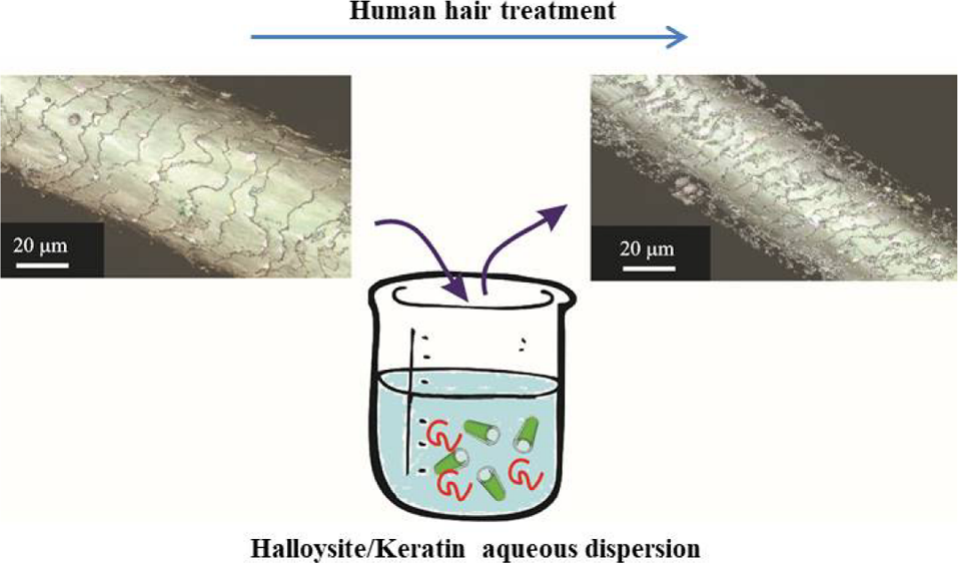

We propose a novel keratin treatment of human hair by its aqueous mixtures with natural halloysite clay nanotubes. The loaded clay nanotubes together with free keratin produce micrometer-thick protective coating on hair. First, colloidal and structural properties of halloysite/keratin dispersions and the nanotube loaded with this protein were investigated. Above the keratin isoelectric point (pH = 4), the protein adsorption into the positive halloysite lumen is favored because of the electrostatic attractions. The ζ-potential magnitude of these core–shell particles increased from −35 (in pristine form) to −43 mV allowing for an enhanced colloidal stability (15 h at pH = 6). This keratin-clay tubule nanocomposite was used for the immersion treatment of hair. Three-dimensional-measuring laser scanning microscopy demonstrated that 50–60% of the hair surface coverage can be achieved with 1 wt % suspension application. Hair samples have been exposed to UV irradiation for times up to 72 h to explore the protection capacity of this coating by monitoring the cysteine oxidation products. The nanocomposites of halloysite and keratin prevent the deterioration of human hair as evident by significant inhibition of cysteic acid. The successful hair structure protection was also visually confirmed by atomic force microscopy and dark-field hyperspectral microscopy. The proposed formulation represents a promising strategy for a sustainable medical coating on the hair, which remediates UV irradiation stress.

## 1 Introduction

Recently, nanotechnology has been explored for novel cosmetic formulations, such as deodorants, nails, anti-aging care, sunscreens, and hair care.^1^ Functional nanoparticles allow for sustained release of drugs with skin penetration enhancing the cosmetic effects. Lipid nanoparticles and liposomes with metal oxides were filled into sunscreen creams.^2,3^ Polymer-based nanosystems were employed as moisturizing and anti-aging skin products.^4^ Chitosan^5,6^ and hyaluronic acid^7,8^ were extensively used for the fabrication of coacervate nanoparticles for cosmetics. Gold^9^ and silver^10^ nanoparticles were incorporated in creams and in deodorants because of their antimicrobial and anti-oxidants anti-aging properties.

Human hair care represents a relevant area involving coloring and medical formulations. Hair color treatments include both permanent and temporary dyes depending on their penetration into the cuticles.^11^ Treatment of dermatological hair diseases caused by lice, fleas, and mites is also important, and nanoparticle encapsulation may increase their efficiency.^12−14^ Finasteride loaded into polymeric nanocarriers was employed to extend protocol against hair loss.^15^ The coating of halloysite clay nanotubes onto the hair surfaces can be exploited to develop efficient coloring and anti-parasite treatments.^13,14,16^ The suitability of halloysite nanotubes for cosmetics is related to their biocompatibility and low toxicity toward both cells^17−19^ and higher organisms.^20−22^ Halloysite nanotubes were employed as controlled drug delivery systems for numerous biomedical and pharmaceutical applications.^23−26^ The combination of natural halloysite nanotubes with biopolymers and biocompatible surfactants allow to obtain green nanocomposite materials.^27−35^ Efficient nanocatalysts were recently achieved by the confinement of ammonia borane within the halloysite lumen.^36,37^

Halloysites are 20–150 nm diameter tubes with 5–70 nm diameter empty lumen capable of loading and slow release of active molecules and proteins.^38,39^ Their length and their specific surface range between 100 and 2000 nm and 28–80 m^2^/g, respectively.^39,40^ It should be noted that the geometrical characteristics and the size polydispersity are affected by the geological deposit of halloysite clay nanotubes.^39,40^ However, typical sizes of commercially available halloysite are of 50–80 nm diameter and 0.5–1 μm length with ca. 60 m^2^/g porosity. The length significantly influences both the drug loading capacity and the release properties of halloysite nanotubes.^17^ Moreover, the mesoscopic structure of biocompatible hybrids is affected by the sizes of the halloysite. As an example, pectin/halloysite nanocomposites evidenced a uniform morphology for shorter nanotubes, while a nest structure was observed in the presence of long and patchy tubes.^17^ Halloysite possesses a surface potential of minus 30–35 mV and a tubes’ aspect ratio of 1/10 to 1/15.^41^ Halloysite has different inner and outer compositions comprising from of alumina and silica groups, and at certain pH in may give a tube with oppositely charged inside/outside surfaces.^42^

The incorporation of active molecules (permethrin and minoxidil) within the halloysite lumen was exploited for sustained hair treatment.^13^ Specifically, permethrin was employed as an anti-lice drug, while minoxidil was used for the alopecia treatment because of its vasodilating properties. According to their hydrophobic nature, both permethrin and minoxidil were loaded into halloysite modified with sodium dodecylsulfate. The hydrophobization of the halloysite lumen can be achieved by the selective adsorption of anionic surfactants on the positive inner surface of the nanotubes.^41^ Halloysite nanotubes were employed as nanocontainers for anionic lawsone (a color component of the traditional Henna herb formulations).^16^ The lawsone entrapment into the clay nanotubes’ lumen was driven by the electrostatic attractions between the anionic dye and, positive at pH 2–8, halloysite internal surface.^41^

We developed protective hair treatment based on the hybrids of halloysite nanotubes and keratin, which is the structural fibrous protein. Keratin is mostly composed of amino acid cysteine, which cross-links through disulfide bonds forming filaments of few nanometer in diameter. The human hair consists of keratin with 18 wt % cysteine content.^43^ Keratin was employed in biocompatible electronic devices,^44^ in hydrogels,^45^ and for nanoparticle formations.^46,47^ As reported in the literature,^48^ polypeptides can repair hair damage because of their capacity to diffuse into the fibers with formation of disulfide bonds and supramolecular interactions between the hair keratin.^48−50^ Recently, the straightening of curly hair was achieved using keratin decapeptides, which were solubilized at neutral water pH.^51^ The interactions between the hair keratin and the repairing peptides are dependent on the pH, which determines the proteins’ charge. In addition, the pH affects the properties of protein/halloysite systems as evidenced for glucose oxidase,^52^ laccase,^53^ insulin,^54^ tyrosine kinases,^55^ and alkaline phosphatase.^56^ Therefore, we studied the pH effect on the structural, thermodynamic, and colloidal properties of halloysite/keratin core–shell systems to optimize the efficiency of proposed hair photo-protection coatings.

## 2 Experimental Section

### 2.1 Materials

Halloysite (HNT) was supplied by Imerys Corp. UK from their Matauri Bay operation. As reported elsewhere,^40^ the average sizes for the halloysite from Matauri Bay are 1000, 80, and 15 nm for the length, outer radius, and inner radius, respectively. In our previous work,^40^ the mineralogical composition of the HNT sample from Matauri Bay was determined through X-ray diffraction analyses, which evidenced the presence of halloysite (87%) and quarts (13%). Hydrolyzed keratin (average molecular weight = 3000 g mol^–1^) was a gift from Kelisema srl. Sodium hydroxide (NaOH) and hydrochloric acid (HCl) are Sigma products. Human hair samples were procured from a healthy Caucasian female, 25 years old with no special treatment.

### 2.2 Preparation of Keratin Solutions in Water

Hydrolyzed keratin solutions at variable pH (from 3 to 12) were prepared by magnetically stirring for 2 h at room temperature. The protein concentration was fixed at 0.2 wt %. The pH of the aqueous solvent was systematically varied by adding proper amounts of 0.1 mol dm^–3^ of NaOH or HCl dropwise.

### 2.3 Preparation of Halloysite/Keratin Aqueous Mixtures

Halloysite nanotubes were added to 0.2 wt % keratin solutions in water at two different pH values (4 and 6). The obtained dispersions were sonicated for 15 min and, subsequently, magnetically stirred overnight at room temperature. The halloysite amount was systematically changed in order to obtain aqueous suspensions with variable halloysite/keratin ratio (from 0 to ca. 0.7).

### 2.4 Human Hair Treatment by Halloysite/Keratin Mixtures

**Figure 1:**
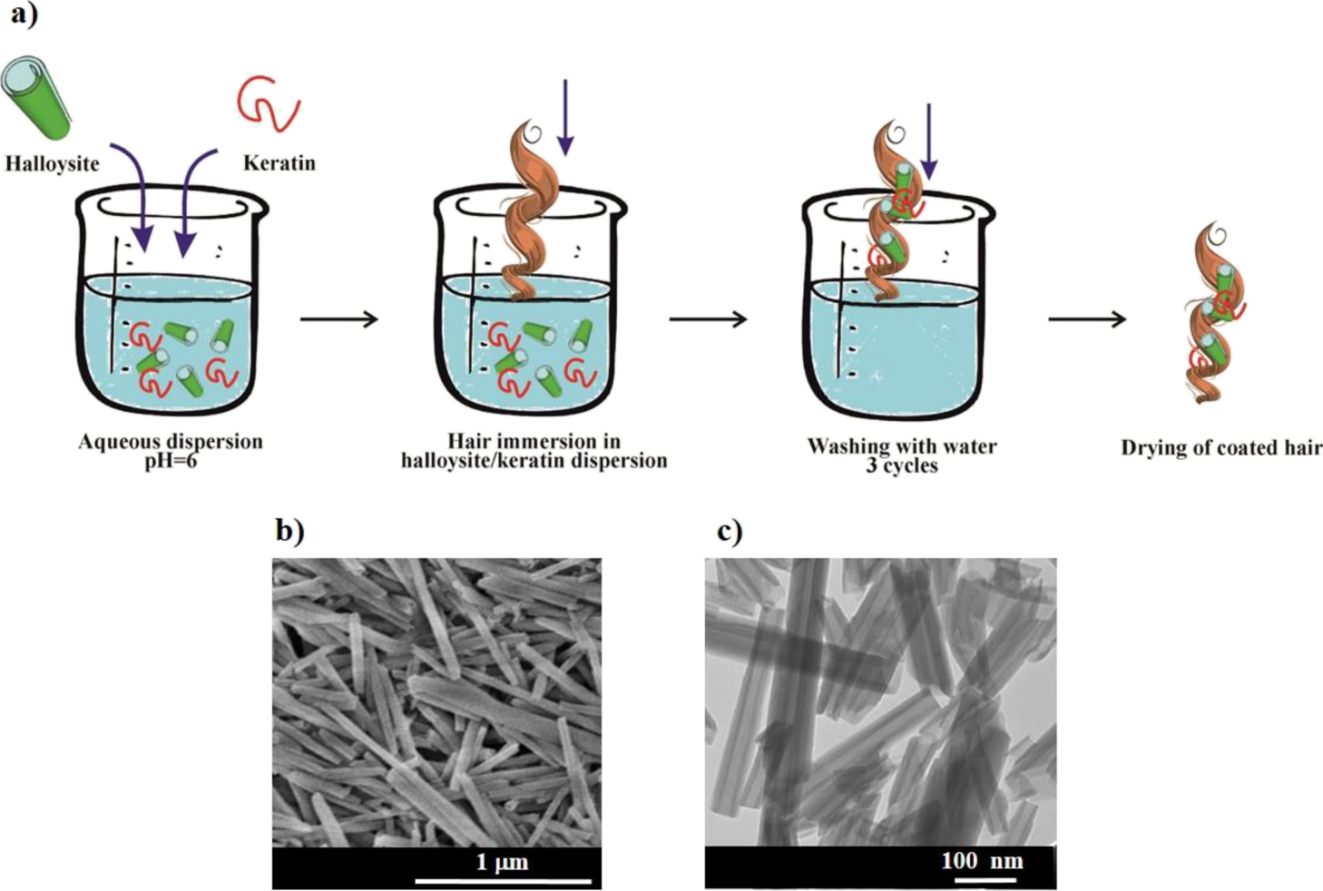
Schematic representation of the hair treatment protocol based on halloysite/keratin aqueous dispersions (a). SEM (b) and TEM (c) images for halloysite clay nanotubes.

Human hair samples were treated by using an immersion procedure in aqueous suspensions. The treatment protocol is sketched in Figure 1. Specifically, hair segments were kept immersed in halloysite/keratin dispersions for variable times (15 and 60 min) to secure partial and full halloysite coating. The pH of the aqueous medium was set at 6, while the halloysite and keratin concentrations were fixed at 1 and 0.2 wt %, respectively. Then, the treated hair samples were washed with water for 1 min. The washing step was repeated three times. Finally, the hair segments were dried at room temperature and they were stored in a desiccator at controlled relative humidity (75 ± 1%) and temperature (25 ± 0.1 °C). For comparison, the described treatment protocol was conducted by using 0.2 wt % keratin solutions in water at pH = 6.

The tubular morphology of the halloysite is shown by electron microscopy images (Figure 1b,c).

### 2.5 UV Irradiation Exposure

Untreated and treated hair segments were subjected to UV radiation from 310 to 400 nm (UV-A region; irradiation equals to 500 W m^–2^). Variable exposure times (24, 48, and 72 h) were investigated.

### 2.6 Methods

#### 2.6.1 ζ-Potential and Dynamic Light Scattering

ζ-potential and dynamic light scattering (DLS) measurements were performed by a Zetasizer Nano-ZS (Malvern Instruments) under isothermal conditions (*T* = 25 °C). For ζ-potential tests, a disposable folded capillary cell was used. Both keratin aqueous solutions and halloysite/keratin aqueous mixtures were investigated. Keratin solutions with a concentration of 0.12 wt % were studied within a wide pH range (from 3 to 12) in order to determine the protein isoelectric point. The mixtures were investigated at a variable halloysite/keratin weight ratio (from 0 to ca. 0.25) under controlled pH conditions. Specifically, pH = 4 (the keratin isoelectric point) and pH = 6 (above the keratin isoelectric point) were selected. It should be noted that the ζ-potential measurements started after a fixed time (5 min of sonication) once the pH of the suspensions reached the selected pH values.

DLS analyses were conducted on keratin/halloysite (mass ratio = 0.25) and halloysite aqueous dispersions. Similarly, to ζ-potential tests, the halloysite concentration was fixed at 0.12 wt %, whereas the pH of the aqueous medium was controlled at two different values (pH = 4 and 6). The field-time autocorrelation functions were fitted through ILT. The wavelength and the scattering angle were kept at 632.8 nm and 173°, respectively.

#### 2.6.2 Differential Scanning Calorimetry

Differential scanning calorimetry (DSC) measurements were performed by means of a micro-DSC III 106 (Setaram) under nitrogen flow under isothermal conditions (*T* = 25 °C) with the aim to investigate the enthalpy of interaction between keratin and halloysite. To this purpose, DSC experiments were conducted using a mixing cell containing 0.20 cm^3^ of halloysite aqueous dispersion and 0.20 cm^3^ of keratin aqueous solution in the lower and upper compartments, respectively. The mixing was initiated by pressing the upper part of the cell. The concentration of keratin was kept constant (0.2 wt %), while the concentration of the halloysite was systematically changed in order to perform calorimetric experiments at variable halloysite/keratin weight ratios (from 0 to ca. 0.7). Experiments were carried out at variable pH conditions. Specifically, pH = 4 and 6 were selected. It should be noted that the effects of dilution of keratin and halloysite were measured and subtracted to the heats of mixing to obtain the thermal effects of the halloysite/keratin interactions.

#### 2.6.3 UV–Vis Spectroscopy

UV–vis spectroscopy measurements were carried out using a Beckman spectrophotometer (model DU-640). The spectra were registered in the wavelength range between 200 and 800 nm at a temperature of 25.0 ± 0.1 °C. The experiments were conducted on keratin aqueous solutions at variable pH (from 3 to 9). The analysis of the absorption peak centerd at 270 nm allowed us to determine the dependence of the specific extinction coefficient of keratin on the pH of the aqueous solvent. As reported for the halloysite/insulin composite,^54^ the loading capacity of the clay nanotubes toward keratin was estimated by UV–vis experiments. The keratin loadings onto the halloysite were determined at pH = 4 and 6.

Moreover, UV–vis spectroscopy was employed to perform turbidimetric experiments on halloysite aqueous dispersions and halloysite/keratin mixtures under controlled pH conditions (pH = 4 and 6 were chosen). Halloysite and keratin concentrations were fixed at 1 and 0.2 wt %, respectively. The sedimentation kinetics of clay nanotubes was investigated by measuring the optical density at 600 nm, where neither halloysite nor keratin exhibit radiation absorption processes.

#### 2.6.4 Atomic Force Microscopy

Atomic force microscopy (AFM) images were collected as described elsewhere^57^ using a Dimension Icon instrument (Bruker) equipped with ScanAsyst-Air (Bruker) probes (nominal length 115 μm, tip radius 2 nm, spring constant 0.4 N m^–1^). Raw data were processed through Nanoscope Analysis software v 1.7 (Bruker). AFM analyses were conducted on the pure halloysite as well as on the halloysite/keratin hybrids obtained by the water evaporation of the corresponding aqueous mixtures, as described elsewhere. Furthermore, AFM analyses were carried out on treated hair samples. Hair segments were attached to glass slides using an adhesive type and imaged under ambient conditions in PeakForce Tapping QNM mode. The top areas along the hair shaft were imaged to minimize the surface curvature interference.

#### 2.6.5 Enhanced Dark-Field Microscopy

Enhanced dark-field (EDF) microscopy analyses were carried out following a published protocol.^58^ Dark-field images at high magnification (100× objective magnification) using a CytoViva EDF condenser attached to an Olympus BX51 upright microscope equipped with a fluorite objective with tunable numerical aperture and a DAGE CCD camera. Extra clean dust-free NEXTERION glass slides and coverslips (Schott, Germany) were used for EDF microscopy imaging to minimize dust interference. We registered the reflected light spectra using a Specim spectrometer and pixelfly USB (PCO) CCD camera. The processing of the spectra was conducted by ENVI 4.8 software. The wavelength interval between 400 and 1000 nm (with a spectral resolution of 2.0 nm) was investigated. EDF images were collected for halloysite/keratin materials suspended in the aqueous mixtures at variable pH values (4, 6, and 8). In addition, human hair was imaged in reflected light dark-field mode supplemented with hyperspectral mapping.

#### 2.6.6 3D Measurement Laser Scanning Confocal Microscopy

A confocal Keyence VK-X150 microscope (equipped with long-distance 100× Nikon objective lens) was used for the morphological investigations of hair surface texture. Hair segments were attached to glass slides, similarly to AFM (as described above). Laser and optical images were processed using VK Analyzer software (v.3.8.0.0) to remove tilt and reduce noise. Morphological analyses were conducted on hair segments treated by keratin and halloysite/keratin. For comparison, confocal images of uncoated hair samples were collected. Surface texture analysis was performed using a Keyence MultiFileAnalyzer software (v.1.3.0.116).

#### 2.6.7 Fourier Transform Infrared Spectroscopy

Fourier transform infrared (FTIR) tests were carried out using a Frontier FTIR spectrometer (PerkinElmer). The spectra were registered in the range between 4000 and 500 cm^–1^ and with a resolution of 2 cm^–1^. The experiments were conducted on KBr based pellets at 25 °C. The effect of the UV irradiation exposure to both uncoated and coated hair samples was investigated by the FTIR spectra analysis. To this purpose, the FTIR region (from 1150 to 950 cm^–1^) related to the cysteine oxidation products was analyzed.

### 2.7 Electron Microscopies

Scanning electron microscopy (SEM) and transmission electron microscopy (TEM) were used for the morphological characterization of pristine halloysite nanotubes. SEM analyses were conducted by a Hitachi S 4800 FESEM microscope at 5–15 kV, while TEM investigations were carried out through a TEM, Zeiss EM 912 microscope at 120 kV. SEM and TEM images are presented in Figure 1.

## 3 Results and Discussion

### 3.1 Keratin Solutions: Effect of pH on the Protein Charge

**Figure 2:**
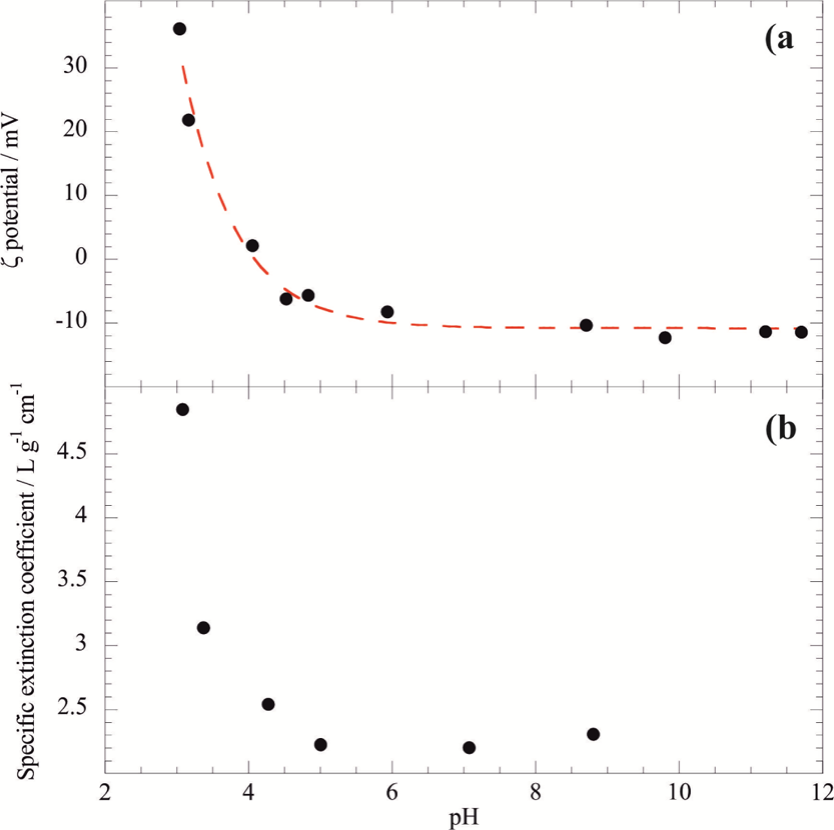
Dependence of the ζ potential (a) and specific extinction coefficient (b) of keratin solution on pH. Dashed red line is the exponential decay approximation.

The keratin aqueous solutions at variable pH were analyzed by ζ-potential and UV–vis spectroscopy measurements. As expected for a protein, the ζ-potential of keratin was strongly affected by the pH of the aqueous solvent (Figure 2a).

The ζ-potential versus pH trend was fitted by an exponential decay equation allowing to estimate the keratin isoelectric point: pH = 4.0. Below the isoelectric point, keratin is positively charged reaching a ζ-potential of 35 mV at pH = 3. Oppositely, keratin exhibited negative ζ-potential at pH > 4.0. Between pH 6 and 12, the keratin ζ-potential is ca. −10 mV. ζ-potential results were supported by UV–vis spectroscopy experiments, which evidenced that the specific extinction coefficient (ε_k_) of keratin is influenced by the pH of the aqueous solvent (Figure 2b); ε_k_ versus pH function exhibited an exponential decreasing trend for pH ≤ 5, and it does not change between pH 5 and 9.

### 3.2 Halloysite/Keratin Aqueous Dispersions: Colloidal Stability and Thermodynamics

#### 3.2.1 ζ Potential and Hydrodynamic Diameter

**Figure 3:**
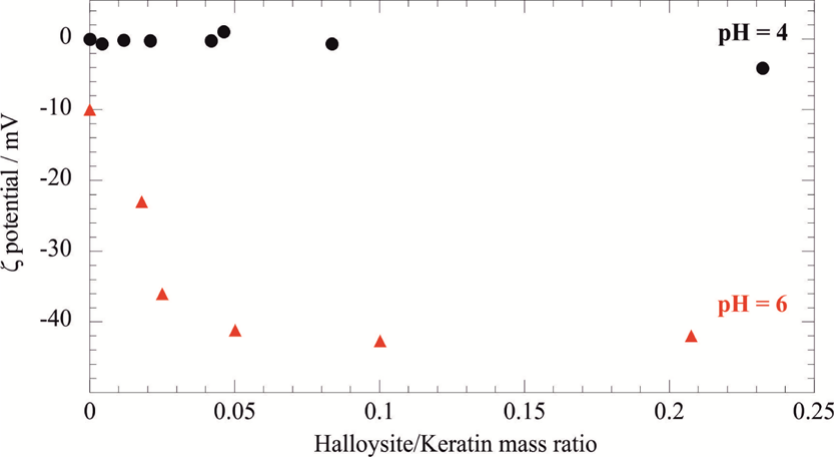
ζ potential as a function of halloysite/keratin mass ratio at pH = 4 (isoelectric point of keratin) and pH = 6 (above the isoelectric point when keratin is negative).

The aqueous colloidal stability of halloysite/keratin dispersions was studied at pH of 4 and 6, where keratin is electrically neutral and negatively charged, respectively (Figure 3).

No electrostatic interactions were found at pH = 4 because the keratin is uncharged (Figure 3, upper dots) and an addition of clay nanotubes did not alter the system ζ-potential. In contrast, attractive interactions between negative keratin and the positive halloysite inner surface at pH = 6 shows a decreasing ζ-potential trend with the increase of halloysite mass until the mass ratio ≤ 0.1, indicating that the saturation of the nanotubes’ surface was reached for the halloysite/keratin mass ratio equals to 0.1. Compared to an electric potential of the pristine natural halloysite of −34.9 ± 0.2 mV at pH = 6, the keratin-loaded nanotubes evidenced an enhancement of the net negative surface charge (−42.9 ± 0.2 mV) that could be ascribed to the neutralization of the positive charges of the halloysite internal surface. Similar effects were observed for halloysite nanotubes functionalized with anionic molecules, including surfactants,^41^ polymers,^59^ and enzymes.^52^ According to the ζ-potential data, the loading capacity of the halloysite toward keratin is larger at pH = 6 (6.37 wt %) with respect to that at pH = 4 (2.47 wt %). Namely, the loading results confirm that the halloysite/keratin affinity is stronger at pH = 6 as a consequence of the electrostatic attractions. It should be noted that the keratin loading at pH = 6 is consistent with the protein confinement within the halloysite cavity, which represents ca. 10 vol % of the clay nanotube.^41^

DLS measurements highlighted that the keratin adsorption does not alter the halloysite hydrodynamic diameter neither at pH = 4 nor at pH = 6. At the keratin isoelectric point, the saturated nanotubes (halloysite/keratin mass ratio = 0.1) possess a hydrodynamic diameter of 720 ± 80 nm, which is close to that of the pristine halloysite (710 ± 80 nm). At pH = 6, the hydrodynamic diameters of the halloysite/keratin hybrid and halloysite are smaller: 590 ± 60 and 620 ± 60 nm, respectively. Accordingly, we can state that the aqueous mobility of the keratin-saturated halloysite is similar to that of pure nanotubes ruling out the formation of nanotubes’ aggregates. Therefore, the aqueous diffusion behavior of halloysite/keratin mixtures at pH 6 is comparable to that of pristine clay nanotubes.

The influence of pH on the hydrodynamic diameter of halloysite/keratin hybrids can be interpreted on the basis of the ζ potential data (Figure 3) by taking into account that the aqueous diffusion coefficient is affected by the repulsive interactions occurring between the nanotubes dispersed in water. The smaller hydrodynamic diameter at pH = 6 (590 ± 80 nm) reflects the faster aqueous diffusion compared to that at pH = 4 (hydrodynamic diameter = 720 ± 80 nm) as a consequence of the larger net surface charge, which enhances the repulsions between the keratin-saturated nanotubes.

#### 3.2.2 Kinetics of Halloysite Sedimentation

**Figure 4:**
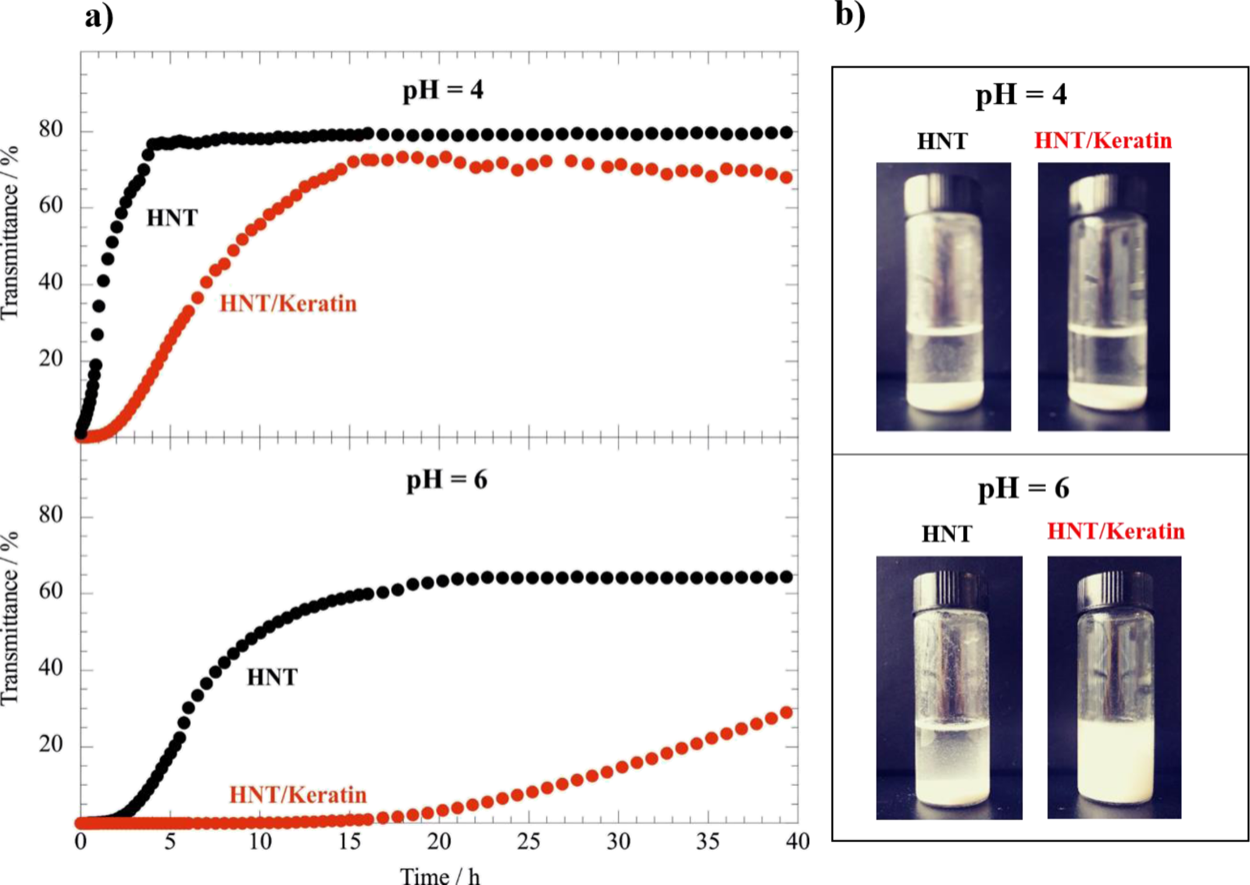
(a) Transmittance at λ = 600 nm as a function of time for halloysite and halloysite/keratin aqueous dispersions at pH = 4 and 6. (b) Photos of the dispersions after 40 h from the preparation. The concentrations of halloysite and keratin were 1 and 0.2 wt %, respectively.

Turbidimetric experiments were conducted on halloysite/keratin aqueous dispersions at different pH in order to estimate the influence of the protein adsorption on the sedimentation kinetics of the composite nanotubes. The transmittance at 600 nm increased over time (Figure 4) because of the sedimentation process, which reduced the concentration of halloysite nanotubes in suspension.

The presence of keratin slowed down the halloysite sedimentation at both pH of 4 and 6 but this is significantly stronger at pH = 6 (Figure 4). This agrees with the ζ potential data at Figure 3, which evidenced that the halloysite/keratin attraction occurring at pH = 6 generate an enhancement of the net negative charge of the nanotubes. Based on the DLVO theory,^60^ the increase of the halloysite charge determines an improvement of its aqueous colloidal stability because of the enhanced repulsive forces occurring between the nanotubes dispersed in water. Similar effects were detected for halloysite nanotubes modified with negatively charged polyelectrolytes and surfactants.^41,61^ Interestingly, an induction time of ca. 15 h was detected in the turbidimetric experiment on halloysite/keratin at pH = 6 (Figure 4). The nanotubes started to precipitate after 15 h highlighting a relevant stabilization induced by the keratin adsorption. In addition, we observed that the suspension is still turbid (i.e., containing suspended nanotubes) even after 40 h confirming the great colloidal stability of halloysite/keratin hybrids at pH = 6.

The sedimentation kinetics fitted the transmittance (*T*) versus time (*t*) trends by the following empirical equation^59^
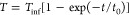
1where *t*_0_ and *T*_inf_ are the characteristic sedimentation time and the level-off value of the transmittance. It should be noted that eq 1 was not successful in the analysis of the sedimentation kinetics of halloysite/keratin at pH = 6 as a consequence of the relevant induction time (15 h). The fitting parameters for the sedimentation process are presented in Table 1.

**Table 1:** Fitting Parameters for the Nanotube Sedimentation Kinetics

aqueous dispersion	pH	*t*_0_/h	*T*_inf_/%
halloysite	4	1.88 ± 0.06	79.8 ± 0.5
halloysite/keratin	4	8.90 ± 0.7	75 ± 2
halloysite	6	10.7 ± 0.8	70 ± 2

We detected that at pH 4 the presence of keratin causes a *t*_0_ increase of five times as well as a slight *T*_inf_ reduction. These provide evidence that the nanotube colloidal stability is enhanced by keratin, which interacts with the halloysite surfaces through supramolecular interactions, including hydrogen bonds and van der Waals forces.

We also observed that pH variation affects the fitting parameters of the halloysite sedimentation (Table 1). In particular, a 4–6 pH increase induced two effects on the fitting parameters: (1) increasing the characteristic time of halloysite sedimentation; (2) reduction of the level-off value of the transmittance. These highlight that the nanotubes possess an improved colloidal stability at pH = 6, consistent with its larger surface charge.

#### 3.2.3 Thermodynamics of Halloysite/Keratin Interactions

**Figure 5:**
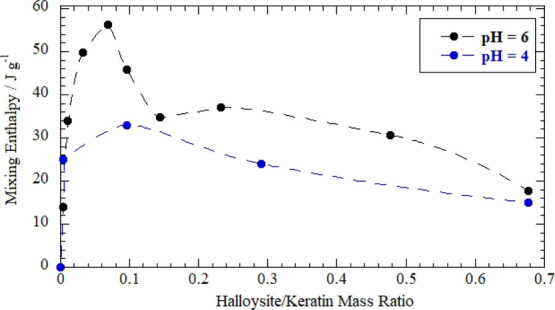
Mixing enthalpy as a function of the halloysite/keratin mass ratio for aqueous mixtures at pH = 4 and 6. The slashed lines interpolate experimental dots.

We studied the keratin adsorption onto halloysite surfaces by DSC measurements under isothermal conditions. We determined the enthalpy of keratin/halloysite mixing in aqueous solvents at pH = 4 and 6. It should be noted the mixing enthalpy (Δ*H*_mix_) was determined by taking into account the dilution effects of both components (keratin and halloysite), which exhibited negative enthalpy values. On this basis, the Δ*H*_mix_ values reflect only the interaction between the two components in water. Figure 5 shows the effect of the halloysite/keratin ratio on the mixing enthalpy, which is expressed as joule per gram of keratin (Figure S1 reports Δ*H*_mix_ values in terms of kjoule per mole of keratin).

The mixing enthalpy is positive indicating that the keratin adsorption onto the halloysite is an endothermic process and therefore the adsorption phenomenon is entropy driven. On this basis, one can argue that the water destructuring as well as counter ion release following the polymer adsorption are the relevant phenomenon contributing to this entropy/enthalpy balance as observed for biopolymer adsorption onto the halloysite.^59^ Moreover, we detected larger Δ*H*_mix_ values at pH = 6 as compared to those at pH = 4, indicating stronger interactions at pH = 6 in agreement with the keratin loading results. The heat generated by the keratin adsorption was affected by the halloysite/keratin mass ratio (Figure 5). In particular, the Δ*H*_mix_ versus halloysite/keratin mass ratio function can be divided in two parts: an increasing trend for halloysite/keratin mass ratio ≤ 0.1, while a further halloysite addition causes the Δ*H*_mix_ reduction highlighting that the additional fraction of nanotubes does not interact with keratin. Namely, the Δ*H*_mix_ decrease can be attributed to the negative dilution enthalpy of the halloysite with water. For pH = 6, the largest Δ*H*_mix_ was observed at the components mass ratio equals to 0.1. This represents the keratin saturation of the nanotubes’ lumen surface in agreement with the Δ*H*_mix_ decreasing trend observed at halloysite/keratin mass ratio > 0.1. Namely, the positive enthalpy due to halloysite/keratin interactions does not contribute to the variation of mixing enthalpy once the saturation point is reached. Calorimetric results are consistent with the ζ potential data (Figure 3), which evidenced that electrostatic attractions between the protein and the nanotubes take place up to their mass ratio ≤ 0.1 and the surface charge of keratin/halloysite hybrids reflects the thermodynamics of interactions between the two components.

### 3.3 Morphological Characteristics of Halloysite/Keratin Hybrids

**Figure 6:**
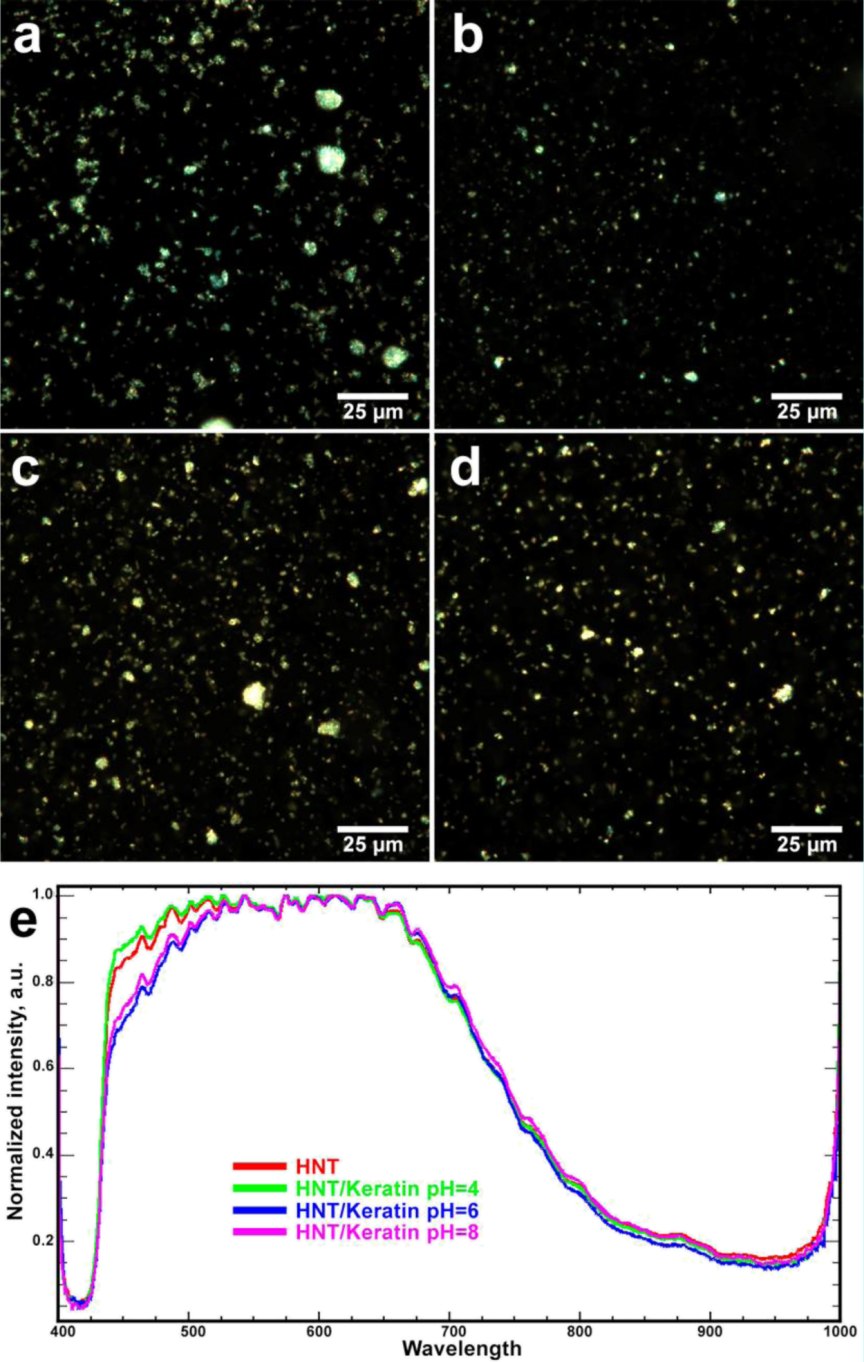
EDF images (a–d) and reflected light spectra (e) for halloysite and halloysite/keratin hybrids suspended in aqueous solvent at variable pH [(a)—HNT; (b)—HNT/keratin pH = 4; (c)—HNT/keratin pH = 6; (d)—HNT/keratin pH = 8].

Deposition of protein-loaded clay nanocontainers onto human hair surfaces for hair protection requires controlling the coating density and the subsequent stability of the coatings to ensure the controllable and predictable behavior of the nanoscale hair coating. Previously, we have investigated the deposition of pristine and hydrophobized halloysites on human hair,^13,14^ here, however, our studies were focused on a different system, a protein-loaded halloysite. Therefore, we investigated the morphological features and nanomechanics of halloysite/keratin hybrids suspended in aqueous solvents at variable pH using AFM operating in PeakForce Tapping nanomechanical mode and dark-field/hyperspectral microscopy. First, to complement the turbidimetry studies, we investigated the colloid stability of halloysite/keratin hybrids using dark-field microscopy at high magnifications (employing 100× oil objective), allowing to visualize individual clay nanotubes in aqueous suspensions.^62^ We imaged pristine and keratin-coated clay nanotubes (Figure 6a–d), which evidenced that the protein adsorption does not induce the formation of large clusters, generally, keratin-coated halloysite nanotubes were well dispersed in water, as well as the pristine nanotubes. This observation is valid for nanotubes coated with both negatively charged (pH = 6 and 8) and electrically neutral keratin (pH = 4).

We also collected reflected light spectra from the pristine and halloysite-coated nanotubes (Figure 6e), which were normalized in visible and near infrared range. The presence of keratin generated a shift to red in the reflected light spectra that can be attributed to the protein adsorption onto the nanotubes. We detected that this effect is reduced at pH = 4, where the keratin coating is not favored by electrostatic attractive forces. Importantly, both the halloysite and keratin-coated halloysite produced a strong light reflection because of intrinsic light scattering efficiency of the halloysite.^63^

**Figure 7:**
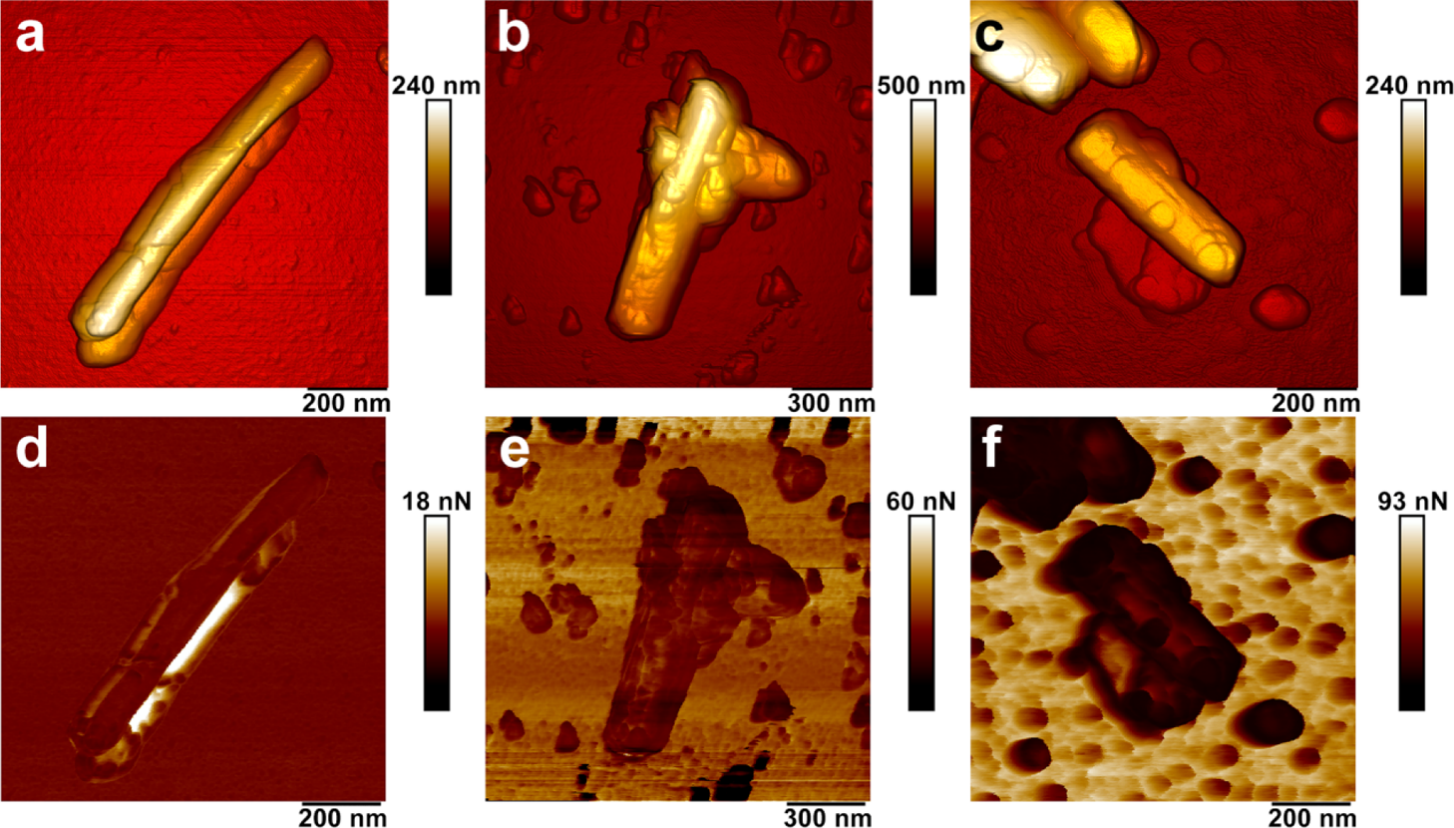
PeakForce Tapping AFM images of halloysite and halloysite/keratin hybrids suspended in aqueous solvent at variable pH [(a,d)—HNT; (b,e)—HNT/keratin pH = 4; (c,f)—HNT/keratin pH = 6]. (a–c)—topography; (d–f)—adhesion).

As we demonstrated previously, the efficacy of hair cuticle coating depends significantly on the surface chemistry of halloysite nanotubes.^13,14^ To further investigate the effects of halloysite loading with proteins on the adsorption rate, we subjected the pristine nanotubes and keratin-loaded nanotubes to PeakForce Tapping nanomechanical AFM in air to investigate their morphology and nonspecific adhesion. Figure 7 compares AFM images of the pure halloysite with those of halloysite/keratin materials dispersed in water at pH = 4 and 6.

We observed the presence of the keratin layer onto the nanotubes confirming the protein coating onto the halloysite surfaces. In addition, we measured the nonspecific adhesion between the keratin-coated halloysite particles and silicon nitride tip of the AFM probes. Pure halloysite has displayed a relatively low adhesion force (3 ± 0.5 nN), which increased twice (6.3 ± 2.1 nN) in the case of halloysite coated with keratin at pH 4 and almost 5 times (14.6 ± 5.9 nN) in the case of halloysite coated with keratin at pH 6. We assume that the increase of nonspecific adhesion force is stimulated both by the deposition of keratin and its spatial conformation because of the pH value during deposition. This suggests that the keratin-coated halloysite will effectively adhere to hair cuticles (which will be shown later).

### 3.4 Treatment of Human Hair by Halloysite/Keratin Aqueous Mixture

Halloysite/keratin aqueous dispersions were investigated for the treatment of human hair. To this purpose, halloysite/keratin mixtures were prepared in aqueous solvents at pH = 6 because of their higher adhesion, relevant colloidal stability highlighted by the ζ potential results and the sedimentation kinetics. Morphological investigations allowed us to estimate the hair coating efficiency, which was correlated to the UV protective action of halloysite/keratin. For comparison, the hair samples were treated with keratin solutions using the same protocol employed with the halloysite/keratin mixtures.

#### 3.4.1 Surface Texture of the Keratin/Halloysite Coated Hair Cuticles

**Figure 8:**
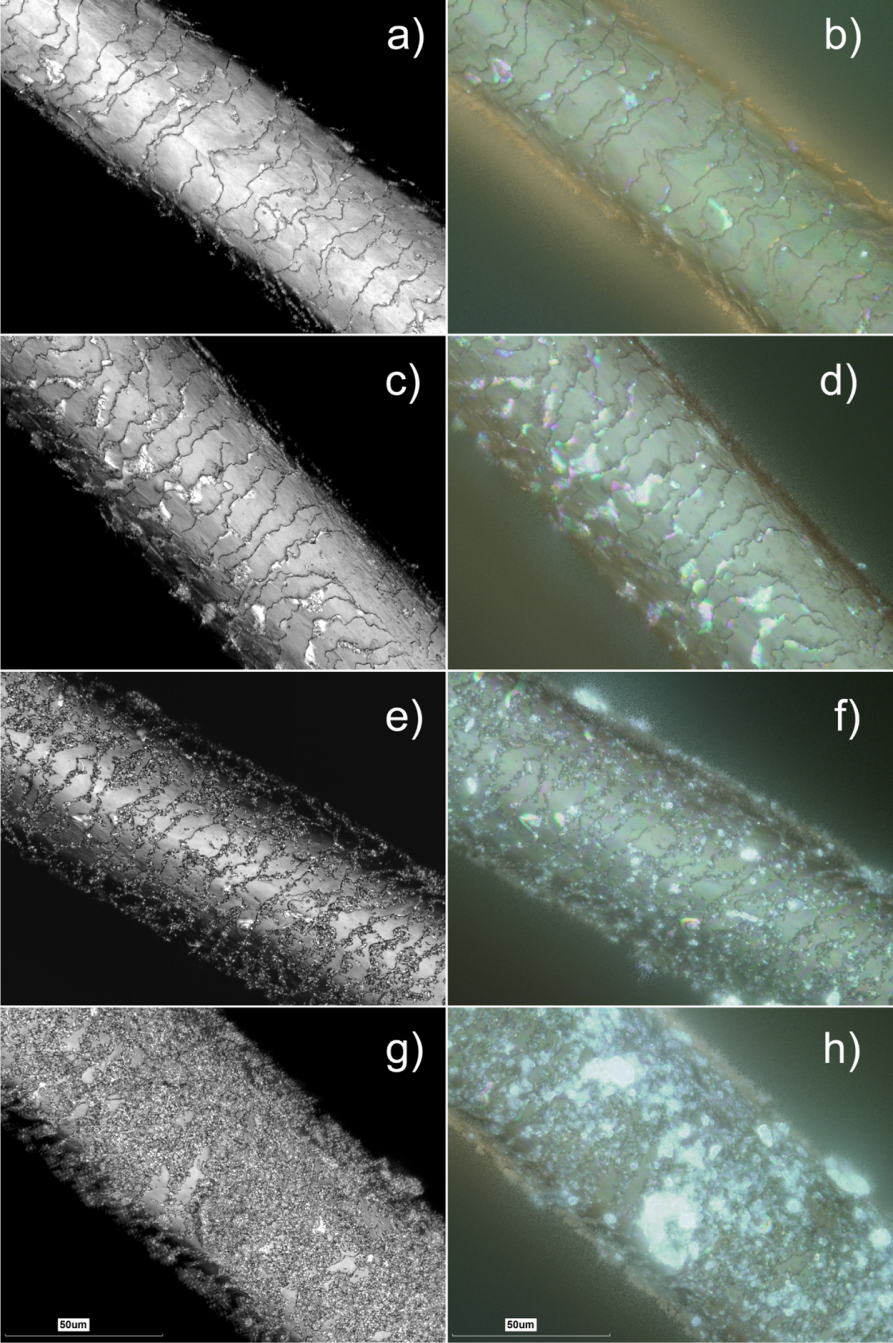
3D measurement laser scanning microscopy of untreated hair (a,b), hair treated by keratin solution for 60 min (c,d), hair treated by the halloysite/keratin mixture for 15 (e,f) and 60 min (g,h). Every left image in each row was obtained as laser intensity grayscale image, while every right image represents an optical reconstruction (in real colours) of the same area.

As we reported previously,^13,14^ the deposition of halloysite nanotubes on mammal hair can be effectively visualized using either SEM or 3D measurement laser scanning microscopy. Although SEM images provide better resolution, here we resorted on using 3D measurement laser scanning microscopy because of two reasons: (i) the samples can be images “as is”, without gold or carbon sputter-coating, which allows evaluating the real color of coated hair and (ii) height (*Z* scale) data obtained can be analyzed for numerical surface structure evaluation with nanoscale precision. Therefore, the morphological features of pristine and treated hair samples were investigated by 3D measurement laser scanning confocal microscopy (Figure 8). We have specifically investigated human Caucasian hair coated with (i) hydrolyzed keratin (which is a popular additive in various hair care products and (ii) keratin/halloysite hybrids (at two exposure times: 15 and 60 min).

We have chosen these exposure times as realistic exposures which can be used for hair treatment. Hair samples (having diameters 61.2 ± 5.1 μm) were positioned diagonally on glass slides and images in superfine mode. Laser intensity images were obtained along with optical and height data, the latter was used to obtain surface texture parameter values. As shown in Figure 8, the treatment by keratin solution did not induce any significant variations on the visible surface morphology of the hair segment, although keratin deposited can be clearly seen as glossy light-reflecting areas on hair cuticles. In contrast, the well-developed surface coating was clearly seen in the hair samples treated by halloysite/keratin mixtures (Figure 8e–h). According to the literature,^13,14,16^ we observed that the cuticles represent the anchoring sites for the hair coating by halloysite nanotubes (which will also be demonstrated later in hyperspectral images). This observation refers to the sample treated for 15 min (Figure 8e,f), where the hair surface is only partially covered by the nanotubes, and the rows between the hair cuticles are primarily targeted by the keratin/halloysite hybrids. Figure 8g,h shows that a full coverage of the hair surface was achieved after 60 min of treatment by the halloysite/keratin mixture. Interestingly, coating of human hair with halloysite nanotubes loaded with keratin results in increased light scattering from the coated hair manifested in bright areas as seen in the optical counterparts of laser intensity images (which is even better seen in lower magnification 3D measurement laser scanning microscopy images shown in Figure S2 and in dark-field and hyperspectral mapping images shown in Figure S3). Obviously, it is clear that the coating efficiency of the proposed protocol is time dependent up to 60 min of hair immersion in the halloysite/keratin aqueous dispersion. This does not mean, however, that shorter exposure times are not relevant to practical applications, moreover, we suggest that the controllable way of deposition of various components for hair moisturizing, softening, or treatment will be based on partial hair coating in between the hair cuticle.

Further, we investigate the surface texture parameters on the microscale using height data obtained with 3D measurement laser scanning microscopy. Height images were tilt-corrected and subjected to areal surface structure analysis by collecting data within 110 × 25 μm rectangular areas along the hair axis at the top part to minimize any curvature influence. We have used several relevant surface texture parameters, summarized in Table 2.

**Table 2:** Microscale Surface Texture Parameters^a^ in Pristine and Engineered Hair Data

hair sample	Sq/μm	Sz/μm	Ssk	Sku	Sal/μm	Str	Sdr/%
untreated	0.87 ± 0.19	6.17 ± 1.41	–0.66 ± 0.29	2.93 ± 0.41	5.56 ± 0.69	0.1 ± 0.01	0.54 ± 0.22
treated by keratin (60 min)	1.53 ± 0.32	9.85 ± 1.31	–0.80 ± 0.26	3.08 ± 0.36	4.76 ± 0.53	0.086 ± 0.009	2.68 ± 2.98
treated by halloysite/keratin (15 min)	1.30 ± 0.29	9.68 ± 2.34	–0.79 ± 0.27	3.30 ± 0.37	4.96 ± 0.42	0.089 ± 0.008	4.18 ± 2.2
treated by halloysite/keratin (60 min)	1.33 ± 0.33	9.44 ± 1.61	–0.63 ± 0.28	2.90 ± 0.45	4.58 ± 0.39	0.083 ± 0.007	5.60 ± 2.19

a Sq (root mean square height); Sz (maximum height); Ssk (skewness); Sku (kurtosis); Sal (autocorrelation length); Str (texture aspect ratio); Sdr (developed interfacial area ratio).

Deposition of both pure hydrolyzed keratin and keratin-loaded halloysite nanotubes on human hair has led to the increase of areal root mean square height (Sq) and maximum height (Sz) of the hair surface texture. However, the overall height distribution of the hair microscale texture remained unaffected, as demonstrated by skewness (Ssk) and kurtosis (Sku) parameters, indicating the height distribution with respect to the mean profile line and sharpness of peak and valley geometry, respectively. The hair surface before and after deposition of hydrolyzed keratin and keratin/halloysite hybrids demonstrates a deviation of peaks above the main line (as indicated by negative Ssk values) and normal peak and valley distribution (Sku = 3). The decrease of autocorrelation length (Sal) suggests that the surface texture features of keratin and keratin/halloysite hybrids coated hair are finer than in pristine hair, obviously, deposited nanoclays have a much smaller particle size and higher aspect ratio than hair surface cuticles. This observation is further supported by the reduction of the texture aspect ratio (Str) parameter, indicating the increase of directionality in halloysite-coated hair, especially in the case of concentration of halloysite near borders between the hair cuticles. Finally, the increase of the developed interfacial area ratio (Sdr) parameter demonstrates the formation of additional surface area in halloysite-coated hair. Taken together, the results on hair microtopography obtained clearly indicate that the deposition of keratin/halloysite hybrids increases the roughness and interfacial surface area of hair while not affecting the overall geometry and height distribution of hair, apparently because pristine human hair exhibit a very developed and uneven surface.

**Figure 9:**
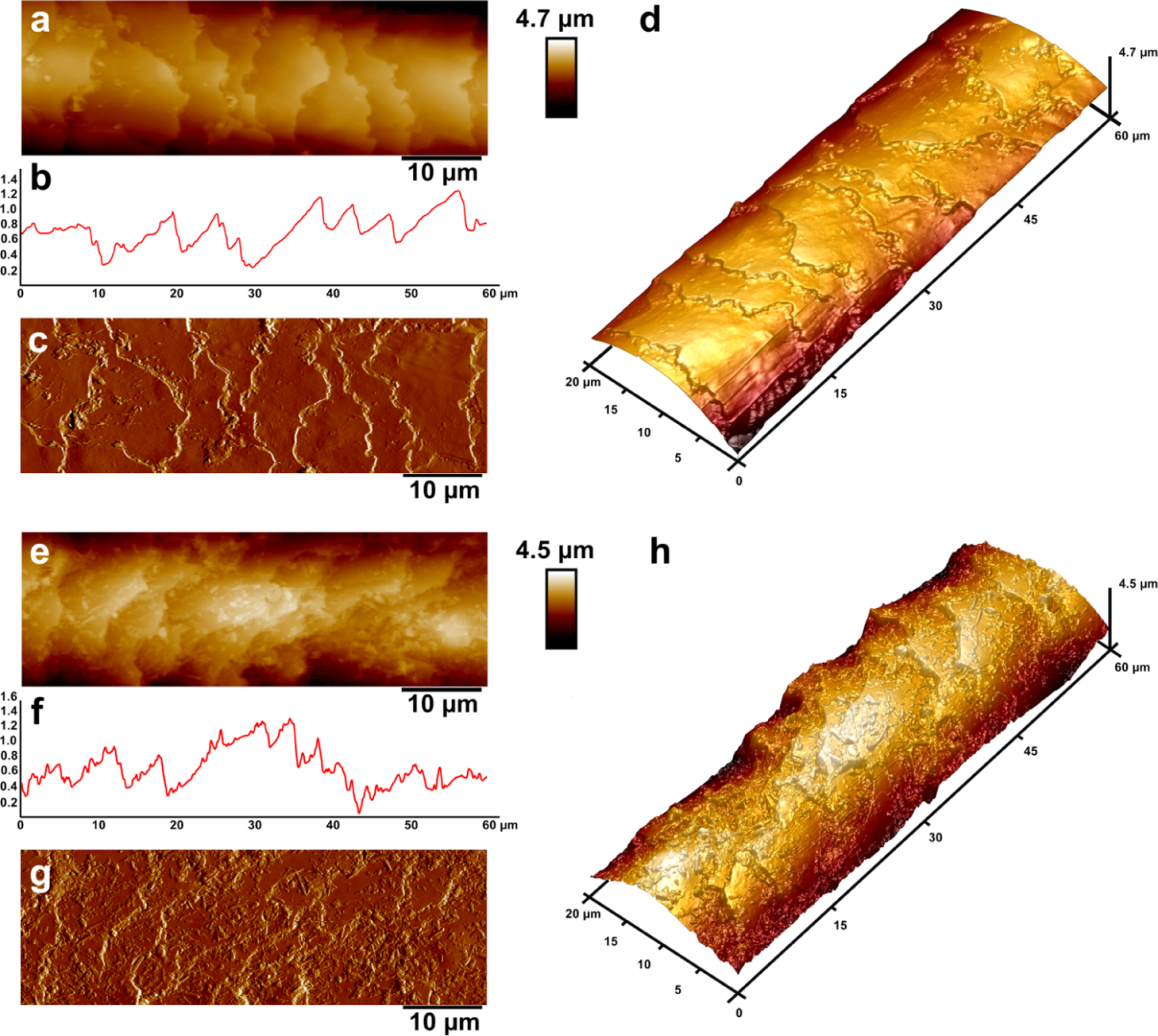
AFM images of pristine human hair (a–d) and keratin/halloysite hybrids coated human hair (e–h): topography (a) and 3D topography (b); surface profile (c) taken across the image shown in (a); PeakForce error image (d); nonspecific adhesion map (e) and Young modulus map (f).

To better understand the surface texture of keratin/halloysite hybrids coated hair we employed AFM, following in part a previously published approach.^64^ AFM is capable of imaging of surfaces at the nanoscale as well as SEM; however, in the case of the former, one does not need depositing a conductive layer on the biological sample, therefore a better representation of an actual surface can be rendered. The second advantage of AFM is the fact that apart from nanoscale imaging one can obtain surface texture parameter values and also nanomechanical data. We used PeakForce Tapping AFM in PeakForce QNM mode to image pristine and keratin/halloysite hybrid-coated human hair in air under ambient conditions. We imaged representative hair fibers having the same thickness to ensure no interference induced by individual hair thickness. The AFM topography and PeakForce error images obtained along with surface height profiles are shown in Figure 9.

The untreated hair (Figure 9a–d) exhibits a typical pattern of undamaged hair cuticles, confirming the healthy status of the hair used in this study. No striations or damaged cuticles (due to destruction of lipid B-layer) were observed. The representative AFM images of keratin/halloysite hybrid-coated human hair (Figure 9e–h) clearly demonstrate the deposition of halloysite nanotubes, which are randomly positioned as a dense monolayer on the cuticles. The line profiles in both samples also confirm the increased surface roughness of halloysite-coated hair, which corresponds well with 3D measurement laser scanning microscopy. Noteworthy, as shown for the AFM topography images, hair cuticles are not damaged by keratin/halloysite hybrids.

We also investigated here for the first time the nanomechanical properties of untreated and keratin/halloysite hybrid-treated human hair employing PeakForce QNM nanomechanical imaging mode. Previously, similar studies were performed in Hybrid AFM mode,^64^ however, arguably PeakForce QNM mode allows for a more precise and straightforward determination of nanomechanical surface mapping in biological samples because of its intrinsic stability and sensitivity, as shown earlier for similar rod-like samples.^65,66^ Specifically, we focused on mapping Young’s modulus and nonspecific adhesion force, the results are shown in Figure S4.

As expected, the keratin/halloysite hybrid-coated hair has become stiffer and demonstrated a higher nonspecific adhesions force, similarly to halloysite nanotubes coated with hydrolyzed keratin. These results confirm that the surface deposition of inorganic nanotubes increase the mechanical stiffness of the pristine human hair, which might be of interest for hair care products industry per se. As for the increased adhesion, this fact is also interesting in view that hair cuticles are built from keratin, however most hair care products utilize hydrolyzed keratin,^50^ which obviously is deprived of the intrinsic 3D structure, which may affect its adhesion. As a result, our composite materials built from hydrolyzed keratin-doped halloysite nanoclay demonstrate excellent adhesion to natural keratin with a well-preserved structure.

#### 3.4.2 Halloysite/Keratin: Hair Protection to UV Irradiation Exposure

**Figure 10:**
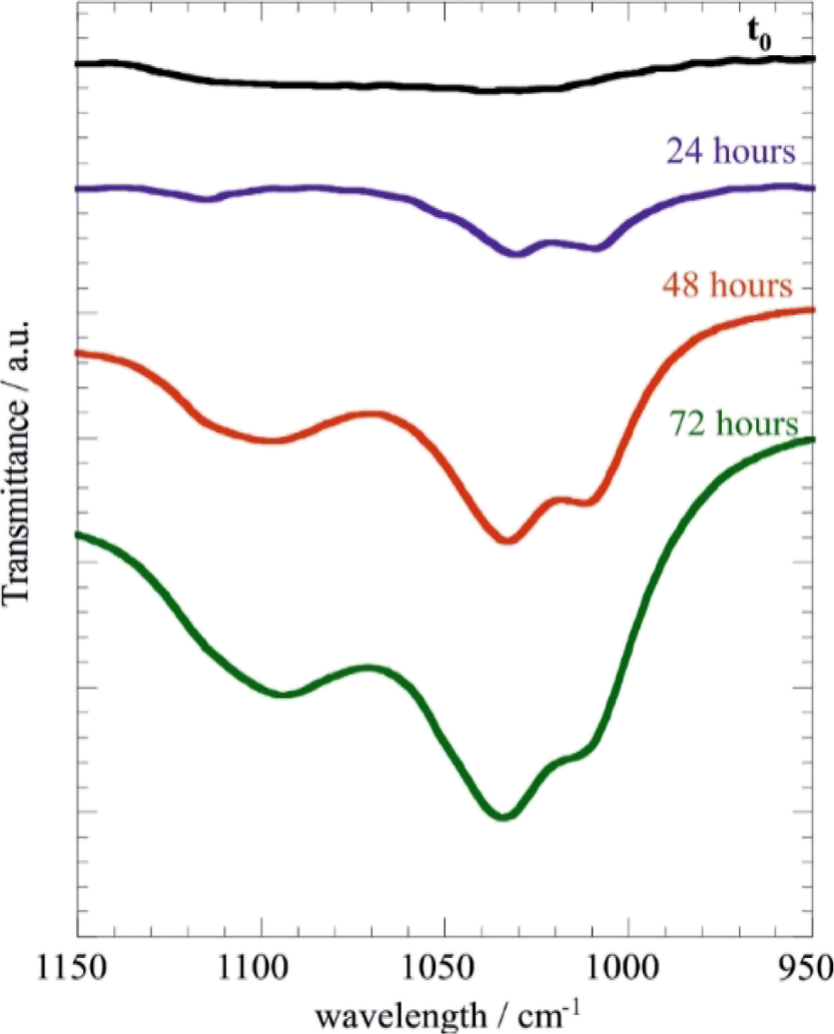
FTIR spectra within the cysteine oxidation region for untreated hair samples exposed UV irradiation at variable time.

The effect of the UV irradiation exposure to the hair structure was estimated by FTIR spectroscopy. In particular, the photo-damage of both untreated and treated hair samples was investigated by monitoring the cysteine oxidation region, which corresponds to the wavelength interval between 1150 and 950 cm^–1^.^67^Figure 10 compares the FTIR spectra of untreated hair exposed to variable UV irradiation time.

We observed that the UV irradiation exposure generates the formation of three FTIR signals, which are related to the products of the cysteine oxidation. According to the literature,^67^ the IR peak centered at 1035 cm^–1^ can be attributed to the stretching of symmetric cysteic acid, while the bands at 1017 and 1092 cm^–1^ are related to cysteine-*S*-thiosulfate and cysteine monoxide, respectively. As expected, the intensities of these signals were enhanced by increasing the exposure time to UV irradiation (Figure 10).

**Figure 11:**
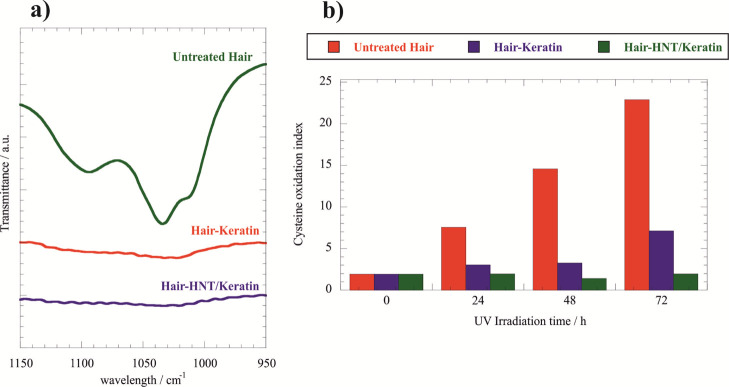
(a) FTIR spectra within the cysteine oxidation region of treated and untreated hair samples exposed to UV irradiation for 72 h. (b) Dependence of the cysteine oxidation index on the UV irradiation time exposure for treated and untreated hair samples.

Figure 11a presents the FTIR spectra within the cysteine region for both untreated and treated hair exposed to 72 h of UV irradiation.

We detected that the formation of the cysteine oxidation products is strongly reduced by both the keratin solution and halloysite/keratin mixture. Accordingly, we can state that the treatment protocol was effective in the hair protection from UV radiation.

In order to estimate the protection efficiency of the treatment, we calculated the cysteine oxidation index by normalizing the peak intensity of the cysteic acid with that of the methylene band at 1450 cm^–1^, which was used as an internal reference. The latter is valid because the CH_2_ deformation is not affected by the photo-oxidation process.^68^ It should be noted that the photo-damage of hair is generally investigated by monitoring the band of cysteic acid, which is formed as a consequence of the cysteine oxidation following the S–S scission.^69^ As shown in Figure 11b, the cysteine oxidation index of untreated hair strongly increased with the UV irradiation time exposure. In this regard, we calculated that the cysteine oxidation index of uncoated hair is one order larger after its exposure to UV irradiation for 72 h. The hair coating with both the keratin solution and halloysite/keratin mixture reduced the effect of the UV irradiation on the cysteine oxidation index (Figure 11b). On this basis, we can state that both keratin and halloysite/keratin hybrids inhibited the formation of the cysteine oxidation products. The UV irradiation for 72 h generated an increase by a factor of 3.5 on the cysteine oxidation index of hair coated with keratin. Interestingly, the cysteine oxidation index of hair treated by halloysite/keratin did not show any variations after UV exposure within the explored irradiation times (24, 48 and 72 h). These results indicate that the halloysite/keratin mixture exhibited a better protection efficiency toward the hair structure with respect to that of keratin solution. Moreover, the halloysite addition extended the protection action time being that the formation of cysteic acid was not detected even after 72 h of UV irradiation exposure.

**Figure 12:**
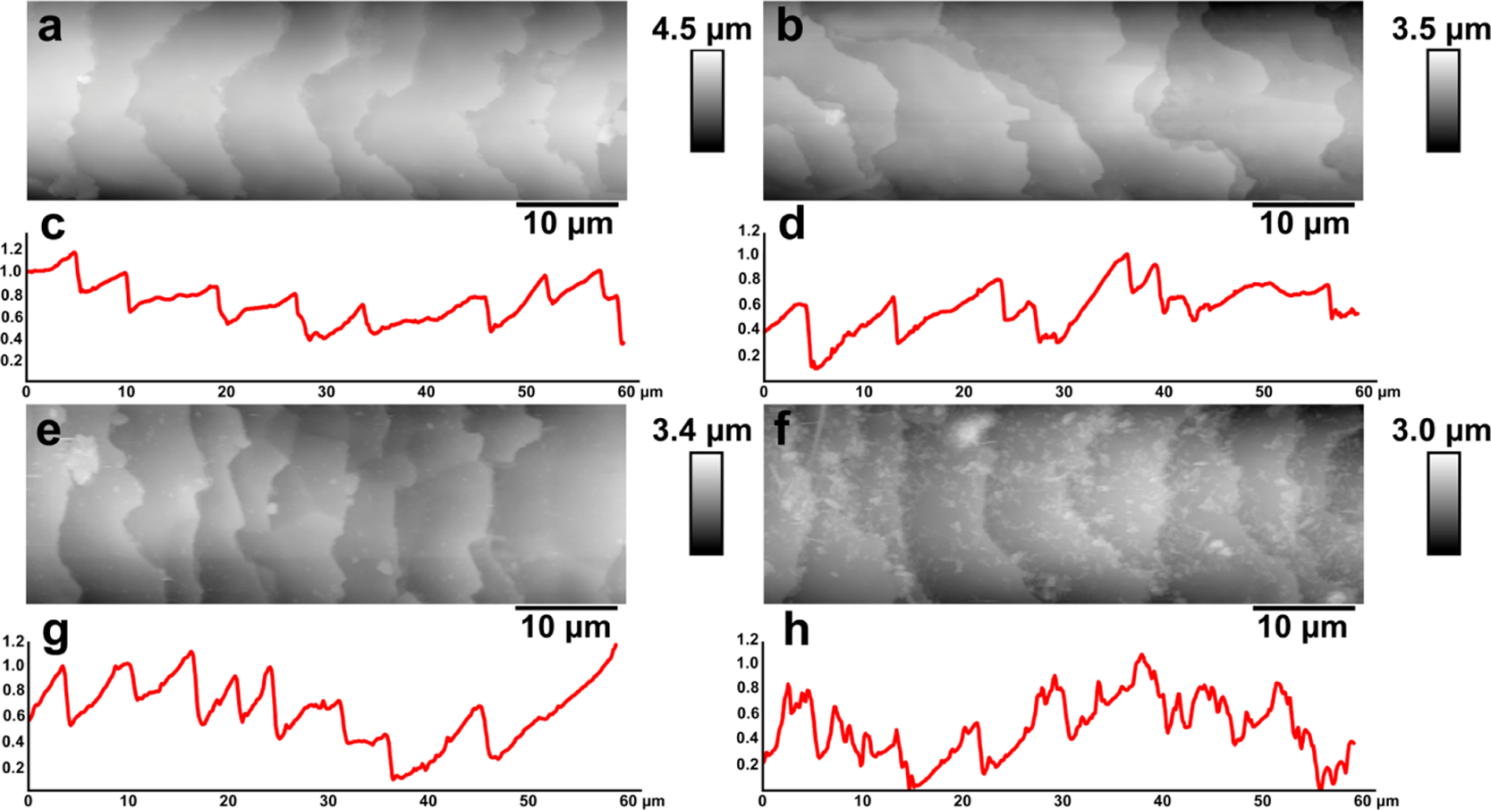
PeakForce Tapping AFM images demonstrating the surface topography and line profile of hair segments: (a) untreated hair; (b) UV-irradiated untreated hair; (c) height line profile for (a); (d) height line profile for (b); (e) UV-irradiated hair treated by hydrolyzed keratin; (f) UV-irradiated hair treated by halloysite/keratin hybrids; (g) height line profile for (e); (h) height line profile for (f). The height line profiles were taken horizontally through the central part of each image.

Figures 12 and S5 show AFM images of treated hair segments after UV irradiation exposure for 72 h.

We observed (Figure S5c) that the hair surface treated by keratin solution is completely covered by spherical structures, which could be attributed to the hydrolyzed keratin globules. The hair coated with halloysite/keratin hybrids evidenced the simultaneous presence of spherical and tubular particles, which represent keratin and halloysite aggregates, respectively. One can see on AFM images and corresponding line profiles that coating of the hair cuticles with keratin/halloysite hybrids improves the hair surface quality upon UV irradiation. To better understand the effects of UV irradiation, we investigated the numerical parameters of hair epicuticles. As shown in Table 3, the cuticle length is only slightly affected by UV irradiation, while the cuticle height is significantly bigger in UV-irradiated hair. Keratin- and keratin/halloysite-coated hair sample exhibit a much bigger cuticle height because of the adlayer formed on the cuticle surfaces.

**Table 3:** Hair Cuticle Sizes

hair sample	cuticle length/μm	cuticle height/nm
untreated	7.5 ± 2.4	326.7 ± 88.5
UV-irradiated and untreated	8.3 ± 4.1	460.6 ± 168.9
UV-irradiated and treated by keratin	7.1 ± 1.9	591.3 ± 143.4
UV-irradiated and treated by halloysite/keratin	9.2 ± 1.6	519.9 ± 110.4

The nanoscale surface roughness (measured as root mean square areal roughness Sq using AFM) on the surfaces of individual hair cuticles (Table 4) suggests that UV-irradiation damages the individual hair cuticles increasing their overall roughness. The increased Sq values for keratin and keratin/halloysite hybrid-coated hair were expected because of the higher asperity of coated hair.

**Table 4:** Nanoscale Surface Roughness in Pristine and Engineered Hair (3 × 3 μm)

hair sample	Sq/nm
Untreated	6.8 ± 4.5
UV-irradiated and untreated	9.2 ± 5.5
UV-irradiated and treated by keratin	12.9 ± 3.2
UV-irradiated and treated by halloysite/keratin	49.3 ± 13.2

Therefore, we believe that the protective effect is caused by both the deposition of hydrolyzed keratin, which provides additional a UV-shielding layer on hair and also by halloysite nanoclay, which renders the hair with high light reflectivity (as shown in Figures S6 and S7, demonstrating the distribution of keratin/halloysite hybrids as imaged in reflected light). As a general result, the hair cuticle structure was not strongly altered by the UV irradiation indicating that the proposed protocol was effective as UV protective coating.

**Figure 13:**
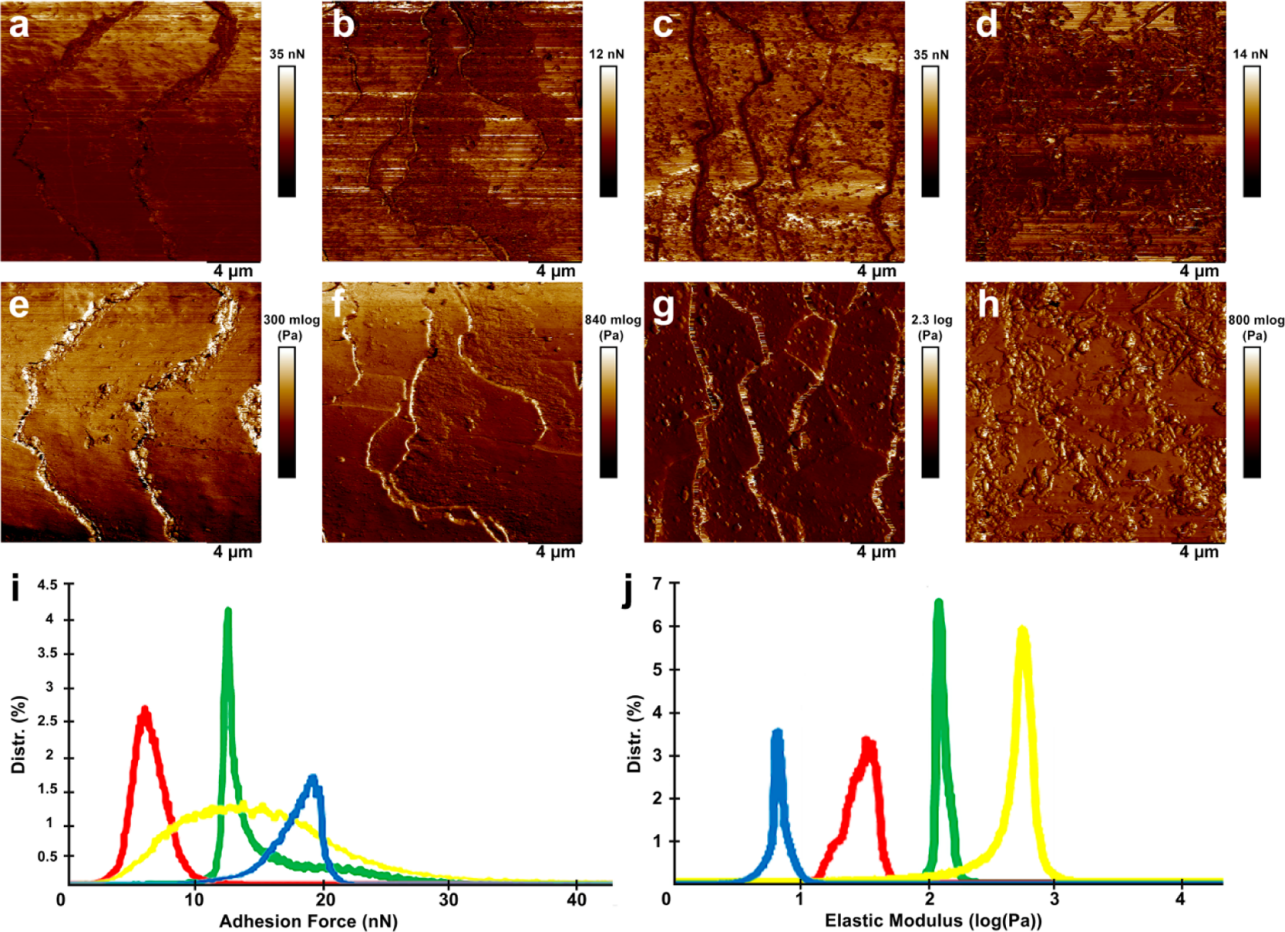
Nanomechanical mapping of human hair segments: (a,e) untreated hair; (b,f) UV-irradiated hair (c,g) UV-irradiated hair treated by hydrolyzed keratin; (d,h) UV-irradiated hair treated by halloysite/keratin hybrids. The images show: (a–d) nonspecific adhesion force; (e–h) Young’s modulus. The distribution of surface adhesion force (i) and Young’s modulus (j): green line—untreated hair; red line UV-irradiated hair; yellow line—UV-irradiated hair treated by hydrolyzed keratin; blue line—UV-irradiated hair treated by halloysite/keratin hybrids.

Furthermore, we investigate the nanoscale mechanical properties of hair cuticles using PeakForce QNM AFM mode. The results are shown in Figure 13.

UV irradiation induces structural changes on the hair surface which manifest via reduction of surface nonspecific adhesion (Figure 13i). In addition, the stiffness of UV-irradiated hair is also reduced if compared to untreated hair (Figure 13j). Obviously, the surface mechanical properties of hair samples coated with either keratin or keratin/halloysite hybrids after UV irradiation cannot be directly compared with those of untreated hair because the deposition of additional layers significantly modifies the properties of hair. It appears that the deposition of pure keratin increases surface nonspecific adhesion and stiffness, while deposition of keratin/halloysite hybrids leads to the increase in surface adhesion while the stiffness is reduced, which we attribute to relative softness of halloysite nanotubes, representing essentially rolled kaolin sheets.^70^

#### 3.4.3 Perspectives of Halloysite/Keratin Mixture for Hair Care Formulations

The halloysite/keratin equilibrium mixture represents a novel hair care formulation with multi-action properties, such as photo-protection and strengthening. Keratin is extensively used in reconstructive masks for damaged hair, while the self-assembly of dye/drug-loaded halloysite nanotubes onto the hair surface was recently exploited for coloring and medical purposes.^13,14,16^ Here, the hair treatment protocol is based on the mixing of the two components in aqueous solvent at controlled pH conditions. Contrarily to previous studies,^13,14,16^ the halloysite was not used as a nanocarrier for functional molecules. In this work, halloysite coating layers enhance keratin binding to the hair providing a long time repairing action. The presence of halloysite nanotubes can reduce the keratin application time (former 3–4 h) and an efficient hair coating of ca. 60% was reached after 1 h of hair immersion in 1 wt % dispersions. Shorter immersion time (15 min) provided a partial hair coating of ca. 30% that can still be sufficient to play an active role, although, the barrier action of the halloysite coating layers is reduced and, consequently, the repairing action is limited to time.

A relevant limitation of the keratin application is represented by hair washing after the treatment. Specifically, hair treated by keratin cannot be washed for up to 4 days in order to guarantee the reconstructive action of the protein. The proposed protocol shows that the halloysite coating is preserved after three hair washing cycles assuring the keratin repairing action. Based on this consideration, the halloysite/keratin hybrid might be added to a shampoo formulation, which possesses pH values between 5 and 7. Nevertheless, it should be noted that the detergents (anionic surfactants) of the shampoo could alter the specific halloysite/keratin interactions and, therefore, the formulation performances.

## 4 Conclusions

Halloysite/keratin hybrids were developed as UV-protective coating layers for surface engineering of healthy human hair. We proposed to immobilize the clay nanotubes on the hair cuticles by using halloysite/keratin suspensions in water. The aqueous halloysite/keratin dispersion optimization in terms of immersion time and pH of the aqueous medium were allowed to maximize the halloysite hair coating efficiency. The halloysite/keratin endothermic interactions are favored at pH > 4 because of the electrostatic attractions between keratin (negatively charged) and the halloysite inner surface, which is positively charged. Interestingly, halloysite/keratin mixture at pH = 6 (optimal condition for efficient hair coating) is stable at least 15 h demonstrating the great colloidal stability of the nanocomposites. Three-dimensional-measuring laser scanning microscopy images highlighted that the coating efficiency is time dependent up to 60 min of hair immersion, and may reach 50–60% of the hair surface coverage using 1 wt % halloysite/keratin dispersion. The protection capacity of the coating layer was explored by monitoring the cysteine oxidation products generated by hair exposed to UV irradiation. The halloysite formulation strongly improved the protective action of keratin to the hair structure, which is visually supported by AFM and dark-field hyperspectral microscopy. In conclusion, the proposed technique can be considered an efficient protocol for the long-term protection of human hair based on natural clay nanotube formulations.

## Supporting Information Available

Mixing enthalpy (expressed as kjoule per mole of keratin) as a function of halloysite/keratin mass ratio for aqueous mixtures at pH = 4 and 6, lower magnification 3D measurement laser scanning microscopy of untreated hair and hair treated by halloysite/keratin hybrids for 60 min, reflected light dark-field optical microscopy image and corresponding hyperspectral mapping of hair treated by halloysite/keratin hybrids for 60 min, nanomechanical mapping of pristine human hair and keratin/halloysite hybrid-coated hair, and higher magnification PeakForce Tapping AFM images, reflected light optical microscopy images (in dark field), and hyperspectral mapping of hair segments before and after UV-irradiation exposure (PDF)
