# Supplementary material for: Halloysite/Keratin Nanocomposite for Human Hair Photoprotection Coating
Source: ACS Appl Mater Interfaces. 2020 May 6;12(21):24348–62. doi: 10.1021/acsami.0c05252 (PMC8007073; doi:10.1021/acsami.0c05252)
Supplement: Supplementary file 1 — am0c05252_si_001.pdf [file am0c05252_si_001.pdf]

Supporting Information

Halloysite/keratin nanocomposite for human hair photoprotection coating

Giuseppe Cavallaro,[*,1,2] Stefana Milioto,[1,2] Svetlana Konnova,[3] Gölnur Fakhrullina,[3] Farida Akhatova,[3] Giuseppe Lazzara,[1,2] Rawil Fakhrullin,[*,3,4] Yuri Lvov[*,4]

[1]*Dipartimento di Fisica e Chimica, Università degli Studi di Palermo, Viale delle Scienze, pad. 17, 90128 Palermo, Italy.* giuseppe.cavallaro@unipa.it

[2]*Consorzio Interuniversitario Nazionale per la Scienza e Tecnologia dei Materiali, INSTM, Via G. Giusti, 9, I-50121 Firenze, Italy.*

[3]*Institute of Fundamental Medicine and Biology Kazan Federal University, Kreml uramı 18, Kazan, Republic of Tatarstan, 420008, Russian Federation.* kazanbio@gmail.com

[4]*Institute for Micromanufacturing, Louisiana Tech University, 505 Tech Drive, Ruston, LA 71272, USA.* ylvov@latech.edu

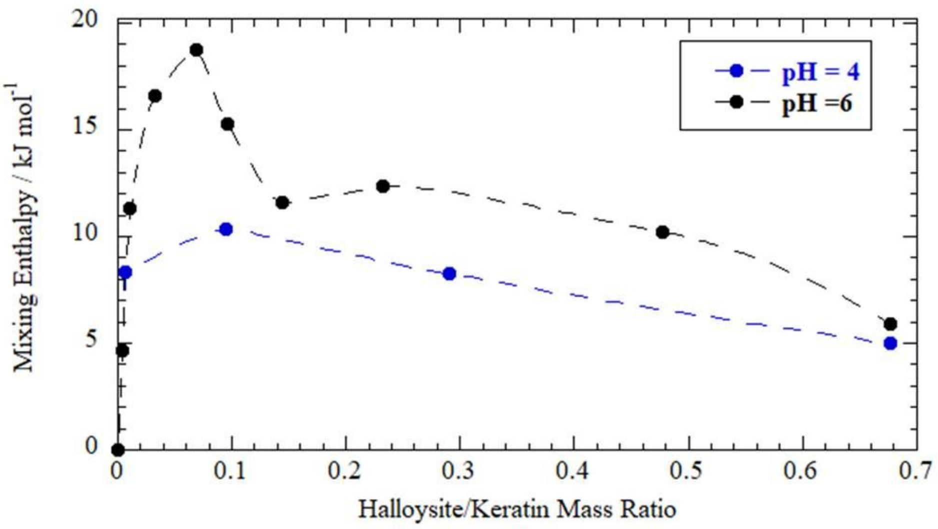

Figure S1. Mixing enthalpy (expressed as kJoule per mole of keratin) as a function of halloysite/keratin mass ratio for aqueous mixtures at pH = 4 and 6. The slashed lines interpolate experimental dots.

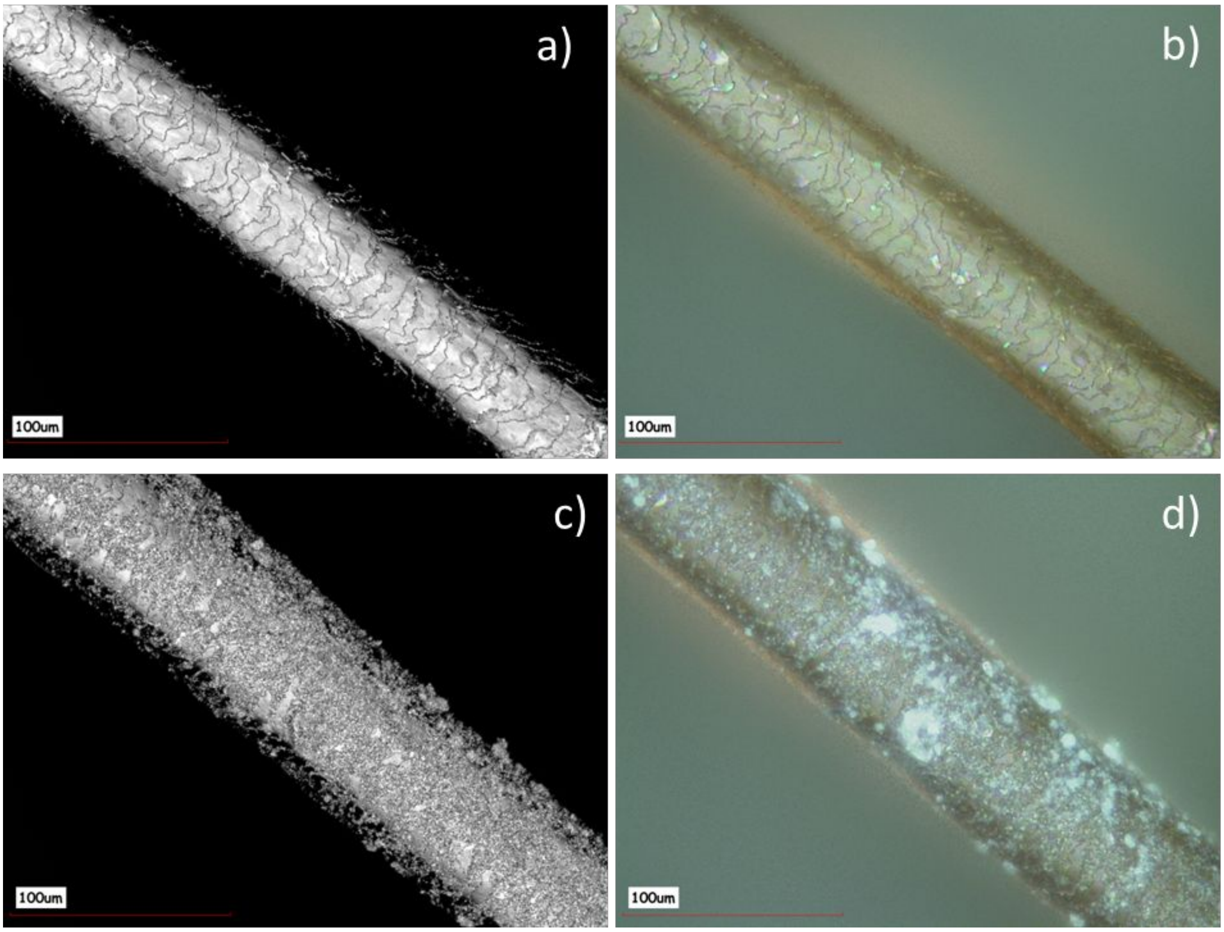

Figure S2. Lower magnification 3D measurement laser scanning microscopy of untreated hair (a-b), and hair treated by halloysite/keratin hybrids for 60 minutes (c-d). Every left image

in each row was obtained as laser intensity grayscale image, while every right image represents an optical reconstruction (in real colours) of the same area.

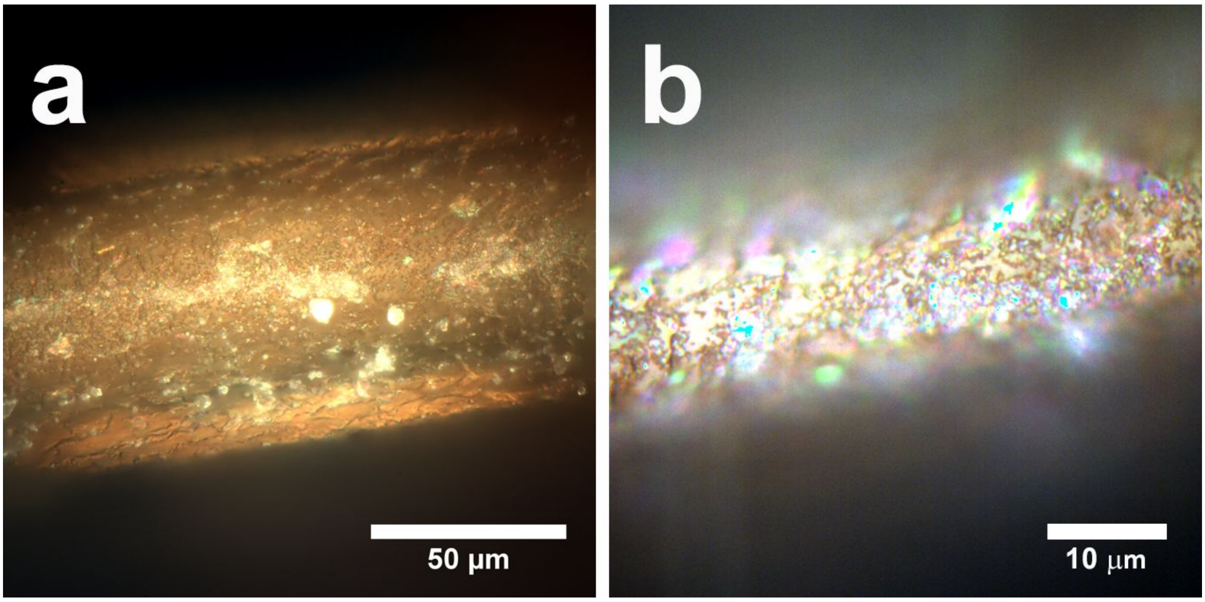

Figure S3. Reflected light dark-field optical microscopy image (a) and corresponding hyperspectral mapping (b) of hair treated by halloysite/keratin hybrids for 60 minutes

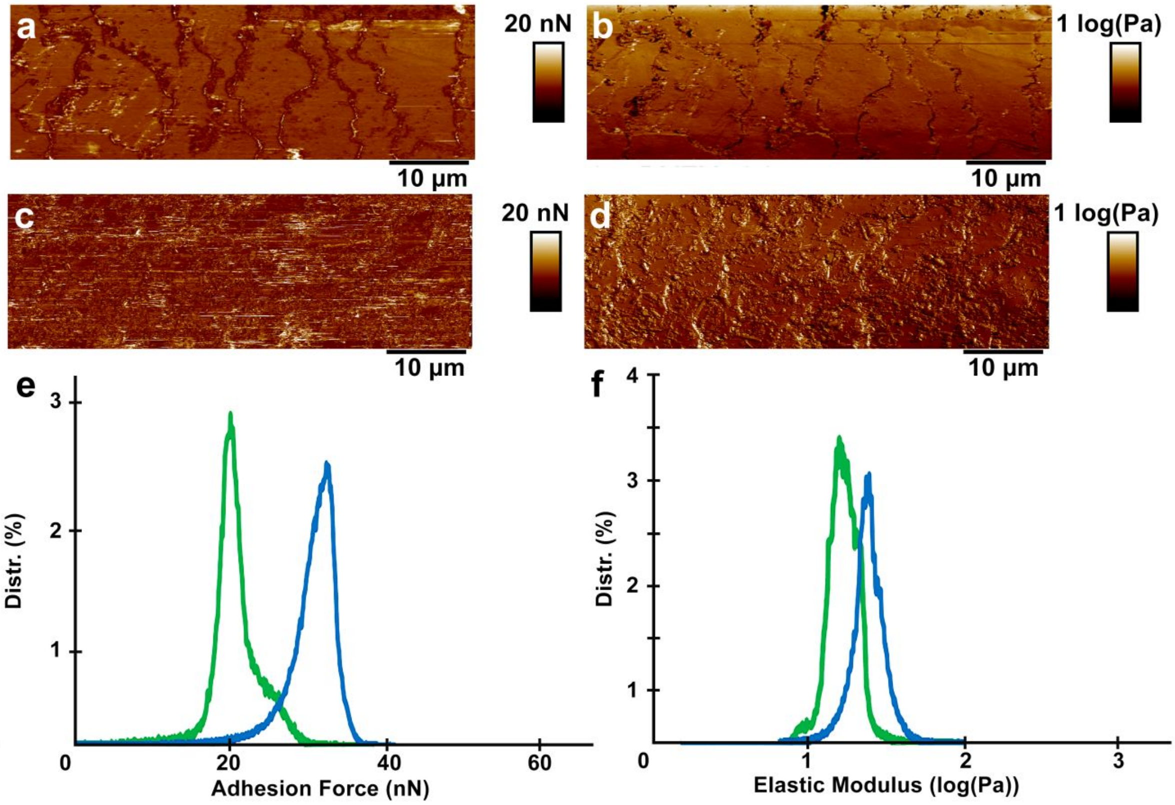

Figure S4. Nanomechanical mapping of pristine human hair (a,b) and keratin/halloysite hybrid-coated hair (c,d), measured as non-specific adhesion force (a,c) and Young's modulus

(b,d). The distribution of surface adhesion force (e) and Young modulus (f), where the green curves correspond to untreated hair and blue curves to keratin/halloysite hybrid-coated hair

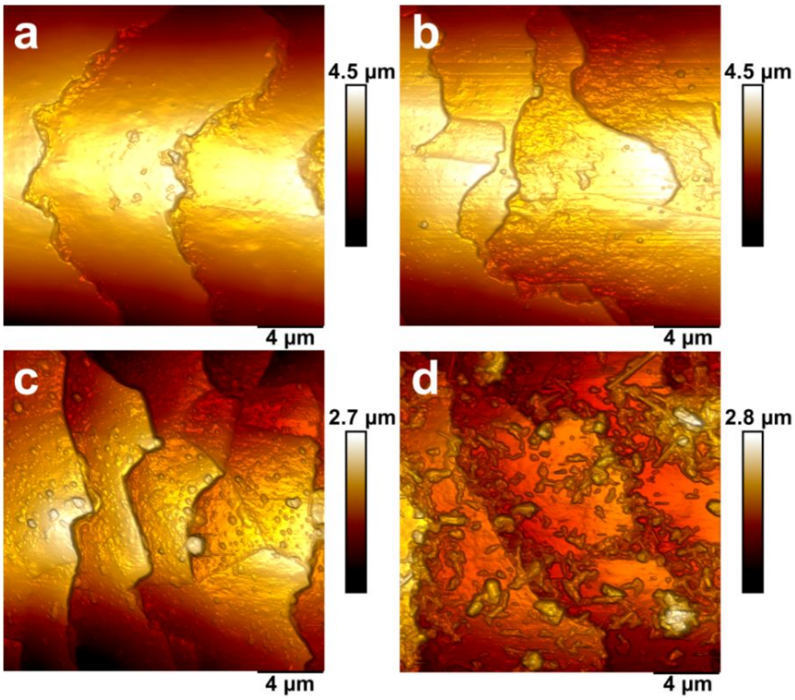

Figure S5. Higher magnification PeakForce Tapping AFM images of hair segments: (a) untreated hair; (b) UV-irradiated untreated hair; (c) UV-irradiated hair treated by hydrolysed keratin; (d) UV-irradiated hair treated by halloysite/keratin hybrids.

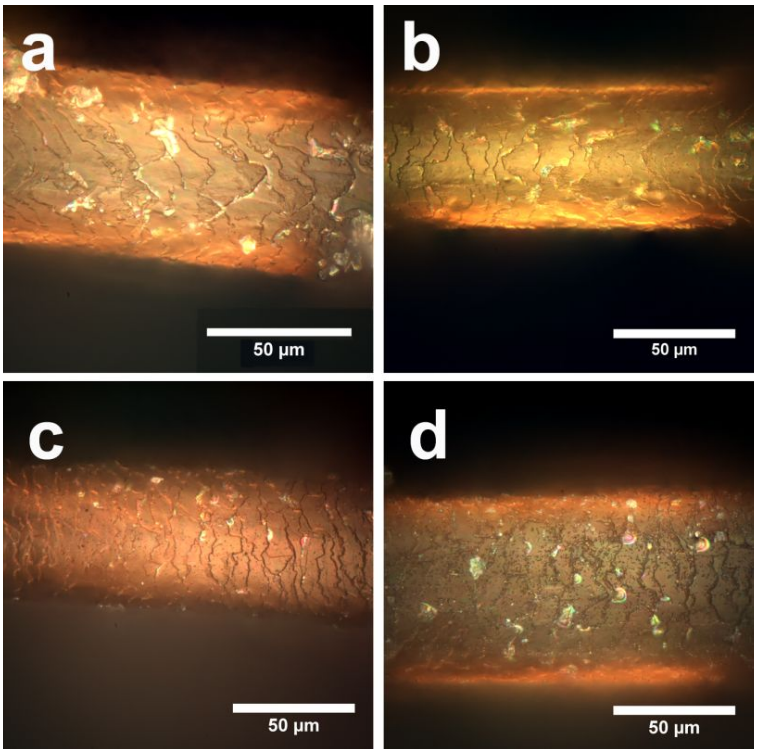

Figure S6. Reflected light optical microscopy images (in dark field) of hair segments: (a) untreated hair; (b) UV-irradiated untreated hair; (c) UV-irradiated hair treated by hydrolysed keratin; (d) UV-irradiated hair treated by halloysite/keratin hybrids.

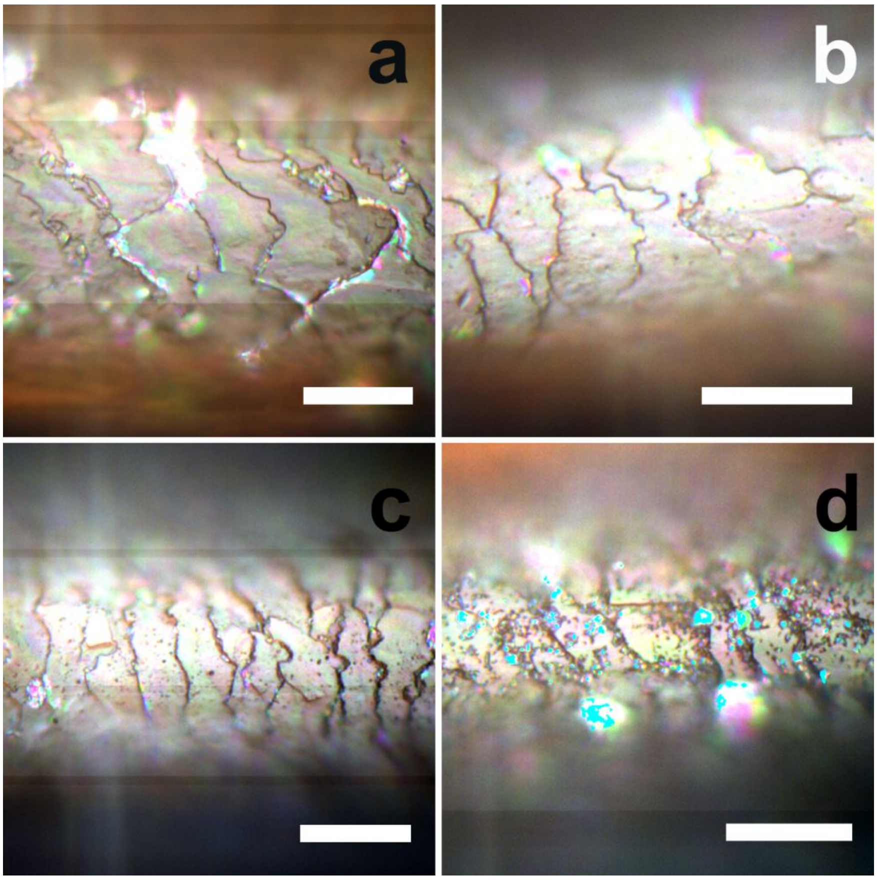

Figure S7. Hyperspectral mapping demonstrating the distribution of keratin/halloysite hybrids on hair segments: (a) untreated hair; (b) UV-irradiated untreated hair; (c) UV-irradiated hair treated by hydrolysed keratin; (d) UV-irradiated hair treated by halloysite/keratin hybrids.
